# Structural insight into PIF6-mediated red light signal transduction of plant phytochrome B

**DOI:** 10.1038/s41421-025-00802-3

**Published:** 2025-05-22

**Authors:** Hanli Jia, Zeyuan Guan, Junya Ding, Xiaoyu Wang, Dingfang Tian, Yan Zhu, Delin Zhang, Zhu Liu, Ling Ma, Ping Yin

**Affiliations:** National Key Laboratory of Crop Genetic Improvement, Hubei Hongshan Laboratory, Huazhong Agricultural University, 430070 Wuhan, Hubei, China

**Keywords:** Plant molecular biology, Cryoelectron microscopy

## Abstract

The red/far-red light receptor phytochrome B (phyB) plays essential roles in regulating various plant development processes. PhyB exists in two distinct photoreversible forms: the inactive Pr form and the active Pfr form. phyB-Pfr binds phytochrome-interacting factors (PIFs) to transduce red light signals. Here, we determined the cryo-electron microscopy (cryo-EM) structures of the photoactivated phyB-Pfr‒PIF6 complex, the constitutively active mutant phyB^Y276H^‒PIF6 complex, and the truncated phyBN^Y276H^‒PIF6 complex. In these structures, two parallel phyB-Pfr molecules interact with one PIF6 molecule. Red light-triggered rotation of the PΦB D-ring leads to the conversion of hairpin loops into α helices and the “head-to-head” reassembly of phyB-Pfr N-terminal photosensory modules. The interaction between phyB-Pfr and PIF6 influences the dimerization and transcriptional activation activity of PIF6, and PIF6 stabilizes the N-terminal extension of phyB-Pfr and increases the Pr→Pfr photoconversion efficiency of phyB. Our findings reveal the molecular mechanisms underlying Pr→Pfr photoconversion and PIF6-mediated red light signal transduction of phyB.

## Introduction

**Fig. 1:**
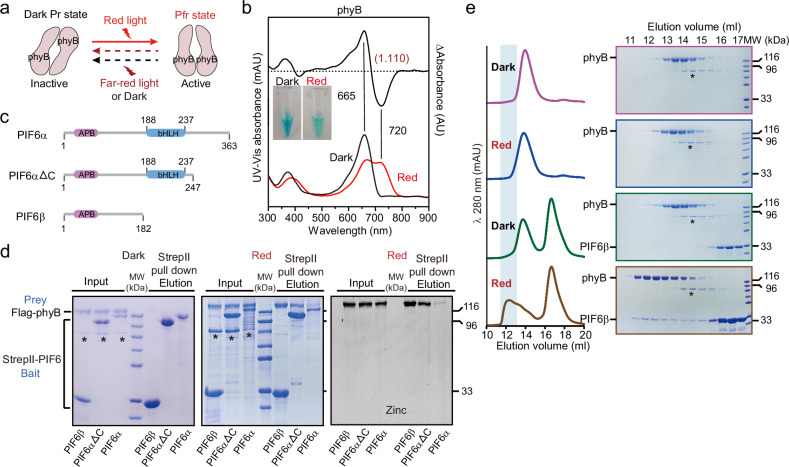
Photoactivated phyB interacts with PIF6 in vitro. **a** In the dark, phyB is in the inactive Pr form. After red light irradiation, phyB converts to the active Pfr form. Upon exposure to far-red light or transfer to the dark environment, phyB-Pfr reverts to phyB-Pr. **b** UV–vis absorbance spectra of phyB in the dark (dark line, Pr) and under red light irradiation (red line, Pfr). The Pr→Pfr difference spectrum is shown at 70% magnitude. The spectral change ratio (SCR) is 1.110. Spectra are the average of three technical replicates. **c** Domain architecture of PIF6. APB, active phyB-binding; bHLH, basic helix-loop-helix. **d** Interactions between PIF6 and wild-type phyB are assessed by pull-down assays in the dark and under red light irradiation. PIF6α, PIF6β, and PIF6αΔC are fused with a StrepII tag. PhyB is fused with 3× Flag tag. Protein mixtures of indicated groups are incubated with Strep-Tactin Sepharose under red light (800 μmol m^−2^ s^−1^) or in the dark for 2 h. Samples in the “Input” and “Elution” are subjected to SDS-PAGE. The proteins are stained with Coomassie blue (left and middle panels), and covalently bound PΦB is characterized by zinc-induced fluorescence (right panel). Asterisks represent nonspecific binding proteins. **e** PIF6β interacts with red light-irradiated phyB in SEC analyses. All SEC analyses are performed using a Superose^TM^ 6 increase 10/300 GL column. Left panel shows the peak fractions of proteins in SEC. The absorbance at 280 nm is detected. The shaded area denotes the peak fractions of red light-irradiated phyB‒PIF6β complex that are co-migrated. Right panel shows the SDS-PAGEs of peak fractions in left panel at the same elution volume from different injections. Asterisks represent nonspecific binding proteins.

Light regulates plant growth and development throughout the entire plant life cycle. Photoreceptors are crucial mediators through which plants perceive light signals^1–3^. Plants have evolved a series of photoreceptors, including the UVB receptor UVB-RESISTANCE 8 (UVR8)^4^ and the blue light receptors cryptochromes (CRYs)^5^, phototropins^6^, and the Zeitlupe/FKF1/LKP2 protein family^7^. Phytochromes (phys) are responsible for the perception of long-wavelength red/far-red light^8–11^. Photoactivated phys regulate various plant developmental processes, such as seed germination, seedling development, shade avoidance, temperature response, stress resistance, secondary metabolite synthesis, and flowering^3,11–15^. The phytochrome family in *Arabidopsis* contains five different phytochrome proteins, namely, phyA, phyB, phyC, phyD, and phyE, of which phyB has been the most extensively investigated^16^. PhyB exists in two distinct photoconvertible forms: the inactive Pr form and the active Pfr form^11,17^. PhyB covalently binds the linear tetrapyrrole PΦB as a chromophore. PΦB contains four pyrrole rings (A‒D), of which the D-ring exhibits a “*Z*” configuration in phyB-Pr. Under red light irradiation, the C15 = C16 bond in PΦB isomerizes into an “*E*” configuration, thus leading to overall conformational changes in phyB from Pr to Pfr. Upon exposure to far-red light or transfer to a dark environment, phyB-Pfr reverts to the phyB-Pr form (Fig. 1a)^11,16–18^.

Since the first molecular identification of plant phyB over 30 years ago, the genetic phenotypes and interaction networks of phyB-Pfr have been extensively examined^11^. PhyB-Pfr translocates into the nucleus^19–22^ and directly interacts with its signal partners^11,23^, including the PHYTOCHROME INTERACTING FACTOR (PIF) basic helix-loop-helix (bHLH) transcription factors, which regulate the transcription of target genes, thereby controlling plant growth, development, and flowering^24–28^. The PIF family in *Arabidopsis* contains 8 members, PIF1‒PIF8, and these PIFs harbor an active phyB-binding (APB) motif at their N-terminus and a bHLH domain at their C-terminus^28,29^. The bHLH domain of the PIFs is subjected to dimerization and binds to the E-box element (5′-CANNTG-3′) within the target promoter DNA^30,31^. Red light-dependent interactions between phyB and PIF3/PIF6 have been reported to be used in optogenetic manipulation^32–38^.

Structural studies of full-length phyB in the form of Pr or Pfr have lagged behind functional studies. *Arabidopsis* phyB (1‒1172) is composed of the N-terminal photosensory module (PSM) (1‒621) and the C-terminal region (655‒1172)^11,39,40^. The PSM module consists of the flexible N-terminal extension (NTE) (1‒110), N-terminal Period/Arnt/Single-Minded (nPAS) domain (115‒219), cGMP phosphodiesterase/adenylyl cyclase/FhlA (GAF) domain (220‒439), and phytochrome-specific (PHY) domain (440‒621). The GAF domain covalently binds PΦB, which is essential for light sensing. The C-terminal region consists of the PAS1 domain (655‒770), the PAS2 domain (800‒905), and the histidine kinase-related domain (HKRD). HKRD is further composed of dimerization histidine phosphotransfer (DHp) (927‒981) and catalytic ATP-binding (CA) (982‒1151) subdomains^11,39,40^. The crystal structure of the PSM of phyB-Pr alone takes on a parallel “head-to-head” dimeric conformation^41,42^, whereas the full-length *Arabidopsis* phyB-Pr forms an asymmetric dimer in which two PAS2 domains mediate a “head-to-tail” assembly of two PSMs. Two PAS2 domains and two PSMs form a parallelogram-shaped platform. The HKRD dimer “sits” at an angle to the platform^39^. During the preparation of our manuscript, cryo-EM structures of phyB-Pfr in complex with PIF6 have been reported. Two PSMs assemble in a “head-to-head” manner^43^. Despite progress in characterizing and understanding the photoactivation of phyB at the structural level, the dynamic process of phyB photoactivation and the ways in which phyB-Pfr and PIF6 influence each other’s functions still require further investigation. In this study, we determined the cryo-EM structures of the photoactivated *Arabidopsis* phyB-Pfr‒PIF6 complex, the constitutively active mutant phyB^Y276H^‒PIF6 complex, and the HKRD-truncated phyBN^Y276H^‒PIF6 complex. A combination of structural and biochemical analyses revealed the molecular mechanisms underlying Pr→Pfr photoconversion and PIF6-mediated red light signal transduction of plant phyB.

## Results

### Photoactivated phyB interacts with PIFs in vitro

We first explored the interactions between phyB and PIF6 via pull-down and size-exclusion chromatography (SEC) assays. In accordance with previous studies^44,45^, we expressed the phyB protein in *E. coli*. The recombinant phyB exhibited maximal absorption at 665 nm in darkness, whereas red light-irradiated phyB displayed maximal absorption at 720 nm, indicating the conversion of phyB from Pr to Pfr (Fig. 1b), which is similar to the findings of a previous report^39^. PIF6 has two splice variants, PIF6α (1‒363) and PIF6β (1‒182)^46^, of which PIF6β lacks the C-terminal bHLH domain. Both PIF6α and PIF6β harbor an APB motif at their N-terminus (Fig. 1c). PIF6β was highly expressed in *E. coli*, but PIF6α was not. We then constructed a C-terminus-truncated form, PIF6αΔC (1‒247, Fig. 1c), and fused it to a large pCold protein tag^47,48^ to improve the solubility of PIF6α. A pull-down assay revealed that PIF6α, PIF6β, and PIF6αΔC interact with phyB under red light irradiation, but their interactions are hardly detectable in the dark (Fig. 1d). Compared with that between PIF6β/PIF6αΔC and phyB-Pfr, the interaction between PIF6α and phyB-Pfr is weak, possibly because proline enrichment at the C-terminus of PIF6α influences the expression and overall folding of PIF6α in vitro (Supplementary Fig. S1a). The SEC assay results corroborated the interaction between PIF6β and phyB-Pfr (Fig. 1e). Moreover, all seven other PIFs in *Arabidopsis* (PIF1‒5, PIF7, and PIF8) contain the APB motif at their N-terminus (Supplementary Fig. S1a, b). A pull-down assay revealed that the N-termini of PIF1, PIF2, and PIF3 interact with red light-irradiated phyB, whereas those of PIF4, PIF5, PIF7, and PIF8 barely interact with it (Supplementary Fig. S1c, d). Considering the optimal behavior and homogeneity of the photoactivated phyB-Pfr‒PIF6β complex among all these recombinant complexes, we further determined its cryo-EM structure.

### Cryo-EM structures of the full-length phyB-Pfr‒PIF6β complex

**Fig. 2:**
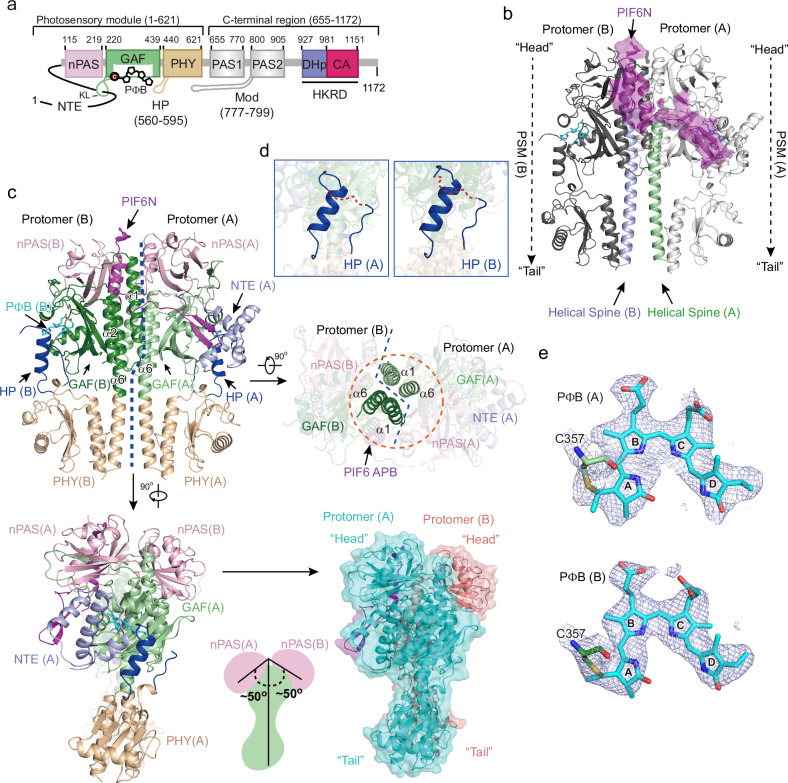
Cryo-EM structures of the phyB-Pfr‒PIF6β complex. **a** Domain architecture of phyB. PhyB contains the PΦB, nPAS, GAF, and PHY domains, the NTE, knot loop (KL), and hairpin (HP) loops within the PSM; the PAS1 and PAS2 domains, the modulator loop (Mod) preceding the PAS2 domain, and the DHp and CA domains within the HKRD region. The numbers represent the start and end points of the domains or loops within phyB. **b** Overall structure of the full-length phyB-Pfr‒PIF6β complex. Two phyB-Pfr molecules (off-white and gray) interact with one PIF6β molecule (magenta). Only PIF6N and phyB-Pfr PSMs are clearly modeled. Two phyB-Pfr PSMs are assembled in a “head-to-head” manner. PIF6N indicates N-terminus of PIF6β (residues 1‒60). **c** Structural overviews of the full-length phyB-Pfr‒PIF6β complex from the front (upper left), top (upper right), and side (lower left) views. The lower right shows the surface of the complex. Domains in protomer A are labeled with “(A)”, and domains in protomer B are labeled with “(B)”. The orange dashed circle represents the four-helix bundle composed of helices α1 and bundle composed of helices α6 from two phyB-Pfr protomers. **d** Hairpin loops (HPs) of two phyB-Pfr protomers. **e** Density maps of PΦB in phyB-Pfr protomer A and protomer B. PΦB adopts an “*E*” configuration, and it is covalently bound to residue C357.

After numerous efforts and several rounds of two-dimensional and three-dimensional classification, we finally reconstructed the EM density map of the phyB-Pfr‒PIF6β complex with an average resolution of 3.1 Å (Fig. 2; Supplementary Figs. S2 and S3 and Table S1; Protein Data Bank (PDB) code: 9JLB). In the phyB-Pfr‒PIF6β complex, two phyB molecules interact with one PIF6β molecule. The PSM (containing the nPAS, GAF, and PHY domains) of each phyB protomer is clearly modeled on the basis of the density map. However, the PAS1, PAS2, and HKRD domains could not be modeled because of the absence of their EM densities. The N-terminus of PIF6β (with only R11‒S60 visible, hereafter referred to as PIF6N) is clearly traced in the EM density map (Fig. 2b; Supplementary Fig. S3), suggesting that the other regions of PIF6β are disordered. The two PSMs from each of the two phyB protomers have a nearly parallel orientation, and they are arranged in a “head-to-head” manner, which is quite different from the “head-to-tail” arrangement observed within phyB-Pr (Supplementary Fig. S4)^39^. Two nPAS domains are suspended at an angle of ~50° on the plane composed of the other domains of the two PSMs (Fig. 2c). Helix 1 and helix 6 (also known as the helical spine) within the GAF domain of protomer A, along with their counterparts in protomer B, form a four-helix bundle to be involved in the dimerization of PIF6β-bound phyB-Pfr (Fig. 2c). PIF6β, which acts analogously to a piece of tape, draws the two phyB-Pfr protomers close together (Fig. 2c). In the structure of phyB-Pr, the NTE is flexible and invisible^39^, whereas in PIF6β-bound phyB-Pfr, the NTE of protomer A interacts with the conserved β sheets of PIF6β and folds into three helices around the PΦB binding pocket (Fig. 2c; Supplementary Figs. S3 and S5). The hairpin loop (HP, also known as the tongue) in the PHY domain folds into an α-helix and is located near the PΦB-binding pockets (Fig. 2d). The A-ring of PΦB covalently links to the conserved cysteine residue C357 in the phyB-Pfr GAF domain, and PΦB adopts an “*E*” configuration, indicating that phyB is in the Pfr state (Fig. 2e; Supplementary Fig. S5). The electron density of the C-ring propionic acid side chains is weak.

In previous studies, the crystal structure of the PAS1-PAS2-HKRD-truncated phyB-Pr PSM showed a “head-to-head” dimerization assembly^41^, which is similar to the “head-to-head” assembly of the PIF6β-bound phyB-Pfr PSM in this study. Despite the similarity, there are some differences in conformations between these two structures, since a series of conformational changes in the PHY domain, the HP loop, and the GAF domain occur during the conversion of phyB-Pr into phyB-Pfr (Supplementary Fig. S4b). Structural superposition of the phyB-Pfr‒PIF6 complex (PDB code: 8YB4 by Wang et al.^43^) with the phyB-Pfr‒PIF6β complex (PDB code: 9JLB in this study) reveals that the overall folds of these two structures are nearly identical (root mean-square deviation (r.m.s.d.) of 0.899 Å over 569 Cα atoms). The PΦB molecules adopt an “*E*” configuration. Both structures demonstrate the α-helical conversion of the HP loop in phyB during photoactivation. PhyB interacts with PIF6 at a 2:1 molar ratio, and only the NTE that interacts with the conserved β-sheets of PIF6 folds into three helices, playing essential roles in PIF6 binding. A pair of β strands (residues 19‒30) and an α helix (residues 46‒60) was clearly modeled in PIF6. The β-strands in the PHY domain slightly differ, possibly due to the resolution of the electron density map in the local region (Supplementary Fig. S6a).

### Cryo-EM structures of the full-length phyB^Y276H^‒PIF6β complex and the HKRD-truncated phyBN^Y276H^‒PIF6β complex

PhyB^Y276H^ has been reported to form photobodies such as phyB-Pfr, shorten hypocotyls, de-etiolate seedlings, and activate the expression of light-regulated genes in darkness^49^, indicating that the phyB^Y276H^ mutant might be constitutively active and possess a Pfr-like structure^50^. The purified full-length phyB^Y276H^ protein exhibited a cyan color in solution, indicating that PΦB bound to this mutant. Unlike that of phyB (Fig. 1b), the absorption peak of phyB^Y276H^ shows little change after red light irradiation (Supplementary Fig. S7a), suggesting that phyB^Y276H^ does not undergo detectable photoconversion under red light, which is consistent with previous reports^41,49^. Furthermore, we explored the interactions between phyB^Y276H^ and PIF6 and found that phyB^Y276H^ interacted with PIF6α, PIF6β, and PIF6αΔC in the dark (Supplementary Fig. S7b, c). Moreover, all seven other PIFs in *Arabidopsis* (PIF1‒5, PIF7, and PIF8) also interact with phyB^Y276H^ in the dark (Supplementary Fig. S7d). These results suggest that phyB^Y276H^ has a Pfr-like structure and is constitutively active in vitro^41,49,50^.

We subsequently determined the structure of the full-length phyB^Y276H^‒PIF6β complex with an average resolution of 2.9 Å (Supplementary Figs. S8 and S9 and Table S1; PDB code: 9ITF). The overall folding of the phyB^Y276H^‒PIF6β complex was nearly identical to that of the photoactivated phyB-Pfr‒PIF6β complex (r.m.s.d. of 0.896 Å over 979 Cα atoms) (Supplementary Fig. S9b). Two phyB^Y276H^ molecules interact with one PIF6β molecule. The chromophore PΦB of phyB^Y276H^ adopts a “*Z*” configuration, which is identical to that of phyB-Pr (Supplementary Fig. S9a). No photoconversion of phyB^Y276H^ was detected under red light irradiation (Supplementary Fig. S7a). In the structure of phyB-Pr^39,41^, the D ring is surrounded by several bulky aromatic residues (Y276, Y303, and Y361; Supplementary Fig. S9b). In photoactivated PIF6β-bound phyB-Pfr, the D-ring of PΦB is in an “*E*” configuration; the side chain of Y303 changes its direction; and Y276 moves away from PΦB (Supplementary Fig. S9b). In PIF6β-bound phyB^Y276H^, the locations and directions of the Y303 and H276 side chains are similar to those in phyB-Pfr. Therefore, residues H276, Y303, and Y361 might stabilize the structure of the PΦB D-ring in phyB^Y276H^. The Y276H mutation induces the HP loop (residues 560‒595) of phyB^Y276H^ to fold into an α-helix (Supplementary Fig. S9c), thus leading to overall conformational changes in phyB^Y276H^. These observations strongly suggest that phyB^Y276H^ adopts a Pfr structure and is constitutively activated.

Considering that the HKRD region is invisible in the structure of the phyB^Y276H^‒PIF6β complex, we investigated whether residues 1‒908 of phyB (hereafter referred to as phyBN) are sufficient for PIF6β binding in vitro^28,29^. We tested the interactions between PIF6β and phyBN^Y276H^, red light-irradiated phyBN, or the HKRD region. Pull-down and SEC results revealed that phyBN^Y276H^ and red light-irradiated phyBN interact with PIF6β, but the HKRD region cannot interact with PIF6β (Supplementary Fig. S10a, b), confirming that phyBN is sufficient for PIF6β binding. Furthermore, we analyzed the phyBN^Y276H^‒PIF6β complex sample using cryo-EM, obtained its density map, and refined it to an average resolution of 2.8 Å (Supplementary Figs. S10c, d, S11 and Table S1; PDB code: 9IRK). Only the PSMs of each phyBN^Y276H^ protomer and the N-terminal region of PIF6β (PIF6N) are clearly modeled, but the PAS1 and PAS2 domains could not be modeled due to the absence of density maps. Structural alignment revealed that the structures of the full-length phyB^Y276H^‒PIF6β complex and the phyBN^Y276H^‒PIF6β complex are nearly identical (r.m.s.d. of 0.657 Å over 966 Cα atoms; Supplementary Fig. S10d). Structural superposition of the phyB^Y276H^-908‒PIF6 complex (PDB code: 9IUZ by Wang et al.^43^) with phyB^Y276H^‒PIF6β (PDB code: 9ITF in this study) revealed that the overall folds of these structures are nearly identical (r.m.s.d. of 0.537 Å over 525 Cα atoms) (Supplementary Fig. S6b). The PΦB molecules adopt a “*Z*” configuration in both structures. The structural alignment results of other regions in both structures are consistent with those of the phyB-Pfr‒PIF6β complexes described in Supplementary Fig. S6a.

### Reassembly of phyB PSMs during PIF6-mediated signal transduction

**Fig. 3:**
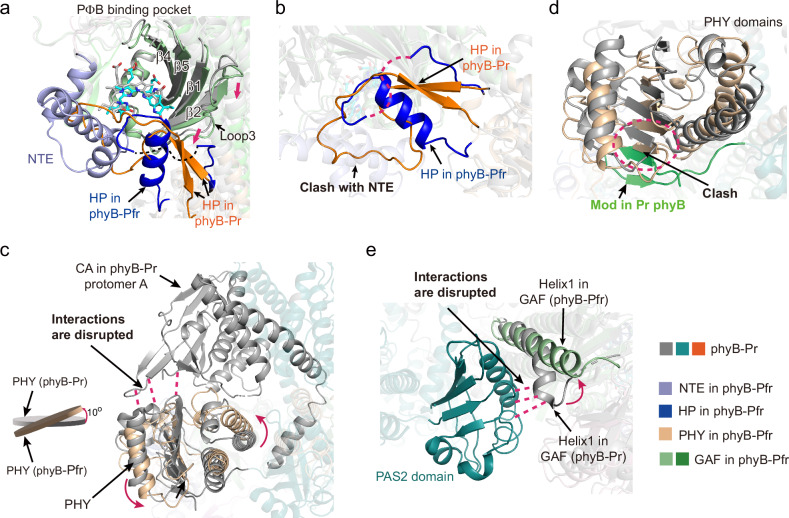
Structural comparison of phyB-Pr (PDB 7RZW) and PIF6β-bound phyB-Pfr. **a** Close-up view of PΦB binding pockets in phyB-Pr (GAF domain, gray; HP, orange) and PIF6β-bound phyB-Pfr. NTE, N-terminal extension; HP, hairpin loop. The missing regions are displayed using dashed lines. **b** Close-up view of HP loops in phyB-Pr (orange) and PIF6β-bound phyB-Pfr (dark blue). **c** Comparison of PHY domains in phyB-Pr (grey) and PIF6β-bound phyB-Pfr (wheat color). The PHY domain in PIF6β-bound phyB-Pfr rotates ~10°, disrupting the interactions (magenta dashed lines) between CA subdomain and PHY domain. CA, catalytic ATP-binding. **d** Modulator loop (Mod) in phyB-Pfr is invisible. Mod in phyB-Pr is green. PHY domains in phyB-Pr and phyB-Pfr are shown in grey and wheat, respectively. **e** Helix 1 of the GAF domain becomes straight in PIF6β-bound phyB-Pfr, disrupting the cross-protomer contacts (magenta dashed lines) between PAS2 domain and GAF domain in phyB-Pr.

The structures of the two phyB protomers in the phyB‒PIF6β complex are highly similar (r.m.s.d. 0.361 Å over 365 Cα atoms; Supplementary Fig. S12). To gain insight into the Pr→Pfr photoconversion of phyB, we aligned protomer A of PIF6β-bound phyB-Pfr with that of full-length phyB-Pr (PDB code: 7RZW). Significant conformational changes are observed mainly at the PΦB binding pockets (**I**), the hairpin loops (HPs) (**II**), the PHY domains (**III**), the modulator loops (Mods) (**IV**), and helix 1 of the GAF domain (**V**) (Fig. 3). Specifically, (**I**) in the PΦB binding pockets of photoactivated phyB, β1, β2, β5, and loop 3 shift to the HP loop, which folds into one α-helix, and the NTE region folds into three α-helices (Fig. 3a, b). The side chains of residues Y104, Y303, Y276, and D307 exhibit changes in orientation (Supplementary Fig. S13). The interactions between loop 3 and the β-sheet of the HP loop disappeared in phyB-Pr, thereby releasing the HP loop. (**II**) Upon release, the HP loop refolds into an α-helix, in which S584 forms a hydrogen bond with D307 (Fig. 3b; Supplementary Fig. S13). In phyB-Pr, the HP loop covers the PΦB binding pocket. In contrast, in PIF6β-bound phyB-Pfr, the HP loop folds into an α-helix, and its original position is occupied by the folded NTE (Fig. 3a, b; Supplementary Fig. S13b). In phyB-Pr, the CA subdomains in the two HKRDs interact with the PHY domain and the GAF domain. The modulator loop (Mod) assembles tightly with the PHY domain. The PAS2 domain in one protomer interacts with helix 1 and helix 6 of the GAF domain in the other protomer. The HP loops from the PHY domains contact the GAF domains and cover the PΦB binding pockets. All these intraprotomer and interprotomer interactions stabilize the PSM “head-to-tail” dimerization assembly mediated by PAS2 domains^39^. (**III**) In PIF6β-bound phyB-Pfr, the α-helical conversion of the HP loop leads to rotation of the PHY domain by ~10°, thus disrupting the interactions between the CA subdomains and the PHY domain or GAF domain (Fig. 3c). (**IV**) The rotation of the PHY domain causes its collision with the Mod (Fig. 3d), hence leading to the dissociation of the Mod and the PHY domain, eventually making the Mod and the PAS2 domain flexible and invisible in the phyB-Pfr‒PIF6β complex. (**V**) In PIF6β-bound phyB-Pfr, helix 1 of the GAF domain becomes straight (Fig. 3e), disrupting the cross-protomer contacts between the PAS2 domain and the GAF domain. All these structural changes result in different dimer interfaces in phyB-Pr and phyB-Pfr. In phyB-Pr, a series of intraprotomer and interprotomer interactions collectively maintain dimerization, including intraprotomer interactions between Mod and PHY, PAS2 and PHY, CA and PHY, and CA and GAF, as well as interprotomer interactions between PAS2 and GAF and DHp and DHp. In contrast, in the PIF6β-mediated phyB-Pfr dimer, there are relatively fewer intraprotomer interactions, with the primary interaction being interprotomer GAF‒GAF interactions (Supplementary Fig. S14).

On the basis of the observed structural changes, we speculate that during the Pr→Pfr conversion of phyB, two PSMs originally assembled in a “head-to-tail” manner under the mediation of the PAS2 domain reassembled in a “head-to-head” manner in the photoactivated phyB-Pfr‒PIF6β complex (Fig. 2; Supplementary Movie S1). To verify this speculation, we performed thiol-directed chemical crosslinking assays to determine whether the PSMs reassemble during Pr→Pfr conversion. We introduced a cysteine mutation, T449C, which is spatially adjacent to the endogenous cysteine (C452) in the other protomer of the PIF6β-bound phyB-Pfr dimer, for specific crosslinking (Supplementary Fig. S15a). According to our hypothesis, after red light irradiation, two PSMs reassemble in a “head-to-head” manner; thus, T449C and C452, which were originally far apart (47.2 Å) in the dark, become much closer (11.4 Å) and can be easily crosslinked by the 1,2-ethanediyl bismethanethiosulfonate crosslinker (Supplementary Fig. S15b). To avoid nonspecific crosslinking, we introduced C925S, C936S, C972S, and C1121S into phyB^T449C^ and phyB^Y276H/T449C^. In the dark, a small amount of phyB^T449C^ is crosslinked. The red light-irradiated phyB^T449C^ and phyB^Y276H/T449C^ are easily crosslinked. The crosslinked phyB^T449C^ and phyB^Y276H/T449C^ can be reversibly reduced by dithiothreitol (Supplementary Fig. S15b). The addition of the PIF6 N-terminal fragment (residues 13‒100) led to a significant increase in the amount of crosslinked bands (Supplementary Fig. S15b). Furthermore, small-angle X-ray scattering (SAXS) analysis was performed, and the results revealed that PIF6β promotes the phyB state transition from Pr to Pfr in solution (Supplementary Fig. S15c). Taken together, the above results collectively suggest that during phyB photoactivation, the PSMs of phyB-Pfr undergo notable conformational changes and are stabilized in a “head-to-head” assembly manner by PIF6.

### PIF6 increases the Pr→Pfr photoconversion efficiency of phyB

**Fig. 4:**
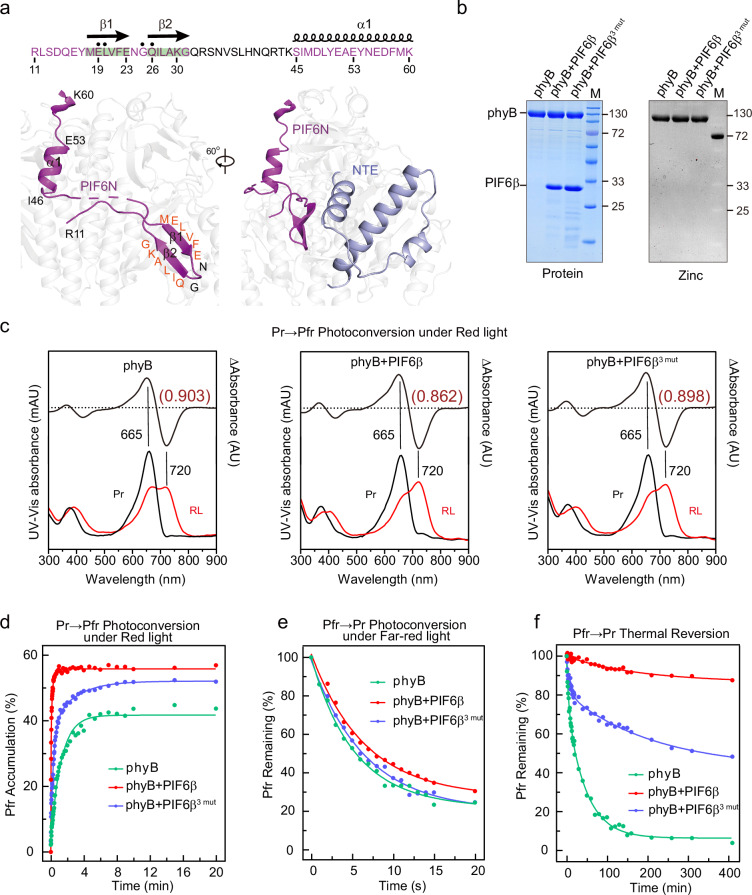
PIF6 increases the Pr→Pfr photoconversion efficiency of phyB. **a** Close-up view of PIF6N structure in phyB-Pfr‒PIF6β complex. The upper panel shows the sequence and the secondary structure of PIF6N. The β1-sheet in the PIF6N interacts with and stabilizes the NTE of the phyB-Pfr protomer A (in light blue). PIF6N denotes N-terminus of PIF6β (residues 1‒60). **b** Proteins used in **c**‒**f** were subjected to SDS-PAGE, and gels were stained for protein with Coomassie blue (left panel) or for covalently bound PΦB by zinc-induced fluorescence (right panel). **c** UV–vis absorbance spectra of phyB alone, phyB + PIF6β, and phyB + PIF6β^3 mut^ in the dark (dark lines) and under red light-irradiation (red lines) are shown. The Pr→Pfr difference spectra are shown at 70% magnitude. The SCRs of phyB alone, phyB + PIF6β, and phyB + PIF6β^3 mut^ are 0.903, 0.862, and 0.898, respectively. Spectra are the average of three technical replicates. PIF6β^3 mut^ indicates a PIF6β mutant which contains E19A, R42A, and I46A triple mutation sites. **d** Pr→Pfr photoconversion of phyB alone, phyB + PIF6β, and phyB + PIF6β^3 mut^ at 25 °C under red light irradiation (617 μmol m^−2^ s^−1^) and monitored at 720 nm. **e** Pfr→Pr photoconversion of phyB alone, phyB + PIF6β, and phyB + PIF6β^3 mut^ with far-red light (655 μmol m^−2^ s^−1^) and monitored at 665 nm and 720 nm. **f** Representative kinetics of Pfr→Pr thermal reversion at 25 °C. The amount of phyB-Pfr remaining after 400 mins is 8 times higher in the presence of the PIF6β. Lines reflect single- or double-exponential kinetic fits as appropriate to the data. Rate constants and amplitudes are provided in Supplementary Tables S2 and S3.

Although PIF6β contains 182 residues, only 51 residues (11‒60, PIF6N) are observed in both the phyB‒PIF6β and phyB^Y276H^‒PIF6β complexes. The SEC assays revealed that PIF6N is sufficient to interact with phyB-Pfr (Supplementary Fig. S16). PIF6N has an extended structure and comprises a pair of β strands (residues 19‒30) and an α helix (residues 46‒60) (Fig. 4a). The β1 (E19‒E23) and α1 (46‒60) regions form a series of hydrogen bonds, salt bridges, and hydrophobic interactions with the NTE and the nPAS domain of photoactivated phyB protomer A and the GAF domain of photoactivated phyB protomer B (Supplementary Fig. S17). The mutational analyses further confirmed the role of these residues in mediating the interactions between phyBN-Pfr (or phyBN^Y276H^) and PIF6N. Mutations Q109A and R110A in phyBN (Q109A, R110A, R177A, and L237A in phyBN^Y276H^), along with mutations E19A, R42A, and I46A in PIF6N, led to a severe decrease in or complete elimination of the interactions between phyBN-Pfr and PIF6N (Supplementary Fig. S18). According to one previous report^28^, the APB motif of PIF6 is subdivided into two more conserved regions separated by a less conserved stretch. Sequence alignment revealed that residues E19, L20, G25, and Q26 in PIF6N are highly conserved across all *Arabidopsis* PIFs (Supplementary Fig. S1a)^28^. In our structure, a β-pair is formed in the first conserved region of the APB motif (residues E19‒E23 and residues Q26‒K30), of which β1 directly interacts with phyB-Pfr. A helix α1 (residues I46‒S60) is formed within the less conserved stretch of the APB motif and interacts with several residues of phyB-Pfr (Fig. 4a), indicating that the less conserved stretch^28^ in the APB motif also plays important roles in phyB-Pfr binding. According to the structural predictions by AlphaFold 2, the N-termini of all seven other *Arabidopsis* PIFs presented a similar structural composition, containing two β-strands and one α-helix, suggesting that PIF members might share a similar phyB-binding pattern (Supplementary Fig. S19).

In the phyB-Pfr‒PIF6β complex, PIF6N interacts with both PSM protomers (Fig. 2; Supplementary Fig. S17). In PIF6β-bound phyB-Pfr, the NTE region of phyB-Pfr protomer A folds into three helices (Fig. 4a), and residues R110 and Q109 in the NTE region interact with residues E19 and L20 in PIF6N, respectively (Supplementary Fig. S17), implying that the NTE region of phyB is essential for PIF6 binding. The folded NTE covers the PΦB-binding pocket (Supplementary Fig. S5a). These observations suggest that the photoconversion efficiency of phyB might be influenced by PIF6β. In the absence of PIF6β, the SCR of Pr→Pfr photoconversion of phyB is 0.903. Upon PIF6β addition, the Pr→Pfr photoconversion efficiency of phyB is slightly improved, as demonstrated by the SCR of 0.862. However, the addition of PIF6β^3 mut^ containing the E19A, R42A, and I46A triple mutation sites (Supplementary Fig. S18) did not significantly improve the Pr→Pfr conversion efficiency of phyB (Fig. 4b, c). The amplitude, Pr→Pfr photoconversion rate constant, and photoconversion quantum efficiency of phyB + PIF6β are all greater than those of phyB alone and phyB + PIF6^3 mut^ (Fig. 4d; Supplementary Table S2). These results suggest that PIF6β increases the Pr→Pfr photoconversion efficiency of phyB under red light. Moreover, we measured the Pfr→Pr photoconversion efficiencies and rate constants of phyB alone, phyB + PIF6β, and phyB + PIF6β^3 mut^ under far-red light or in the dark (thermal reversion at 25 °C). The amount of phyB-Pfr remaining after 20 s of far-red light irradiation or 400 min in the dark in the presence of PIF6β was greater than that of phyB alone or phyB + PIF6β^3 mut^ (Fig. 4e, f; Supplementary Table S3). These results suggest that PIF6β decreases the recovery rate of phyB-Pfr to phyB-Pr under far-red light irradiation or in the dark.

### PhyB influences the DNA binding and transcriptional activation activities of PIF6

**Fig. 5:**
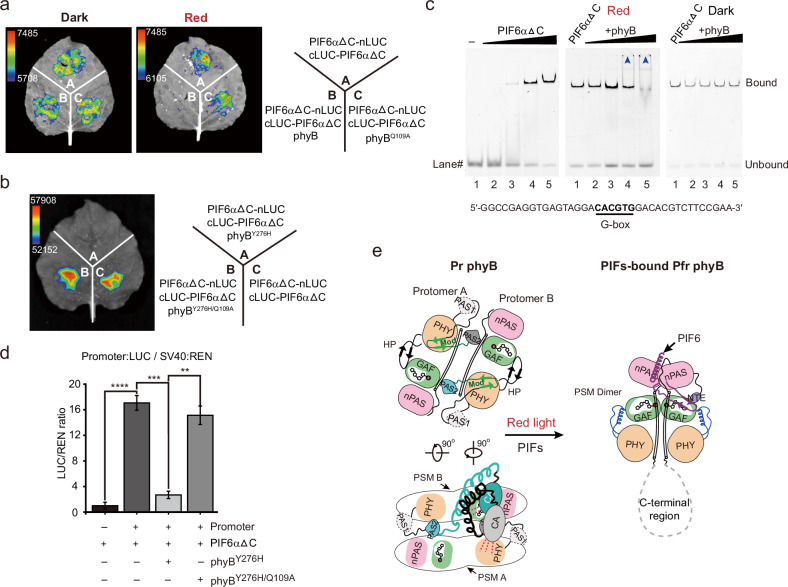
PhyB inhibits the dimerization of PIF6 and influences its DNA-binding and transcriptional activation activities. **a**, **b** LCI assays demonstrate that red light-irradiated phyB (**a**) or phyB^Y276H^ (**b**) inhibits the dimerization of PIF6αΔC bundle composed of helicesΔC in *Nicotiana benthamiana* leaves. Three independent experiments were performed, with similar results. PhyB^Q109A^ and phyB^Y276H/Q109A^ are mutants hardly interacting with PIF6. **c** EMSA analysis shows that PIF6 can bind to the DNA probe containing the G-box (5′-CACGTG-3′) motif. PhyB-Pfr influences the DNA-binding activity of PIF6. A reported G-box-containing DNA probe “G-wt”^30^ is used in the EMSA assays. The DNA probe is labeled with FAM at the 5′ end. Left panel: the final DNA probe concentrations in Lanes 1‒5 are all 15 nM, and the corresponding protein concentrations are 0, 0.375, 0.75, 1.5, and 3.0 μM, respectively. Middle and right panels: EMSA analysis shows that red light-irradiated phyB-Pfr influences the DNA-binding activity of PIF6αΔC bundle composed of helicesΔC. The final concentrations of PIF6αΔC bundle composed of helicesΔC in Lane 1‒5 are all 1.5 μM, and the corresponding concentrations of phyB-Pfr are 0, 0.75, 1.5, 3.0, and 6.0 μM, respectively. **d** Reporter (*FT* promoter: Luc and SV40: Renilla), PIF6αΔC bundle composed of helicesΔC, and phyB^Y276H^ are transfected into the HeLa cells, as indicated by “‒” or “+”. The relative firefly luciferase (LUC) activities are normalized to the Renilla luciferase (REN) activity. *FT*, *FLOWERING LOCUS T*. Data are presented as means ± SD (*n* = 3). **P < 0.01; ***P < 0.001; ****P < 0.0001. PhyB^Y276H/Q109A^ is a mutant hardly interacting with PIF6. **e** A proposed model for the mechanisms of Pr→Pfr photoconversion and PIF6-mediated signal transduction of plant phyB. PhyB-Pfr forms a homodimer in a “head-to-head” manner. PIF6 stabilizes the dimerization of the PSMs and maintains the conformation of phyB-Pfr, and meanwhile, phyB-Pfr inhibits the dimerization and influences the DNA-binding and the transcriptional activation activities of PIF6. Due to the lack of electron density, the structure of C-terminal region (PAS1-PAS2-HKRD domains) in phyB-Pfr requires further investigation.

It has been reported that a homodimer or heterodimer is formed in the bHLH region of PIFs to bind the G-box DNA element^31^. In the structures of the phyB-Pfr‒PIF6β complex and the phyB^Y276H^‒PIF6β complex, phyB interacts with PIF6β at a 2:1 molar ratio (Fig. 2; Supplementary Figs. S9a and S10d). We hypothesized that phyB-Pfr might inhibit the dimerization of PIF6, thereby influencing its DNA binding and transcriptional activation activities. We first conducted a luciferase complementation imaging (LCI) assay in *Nicotiana benthamiana* and fused PIF6αΔC to the N- and C-terminal domains of LUCIFERASE (nLUC and cLUC), respectively. Cotransfection of PIF6αΔC-nLUC and cLUC-PIF6αΔC resulted in robust luciferase activity, indicating the formation of the PIF6αΔC dimer. In the case of the cotransfection of phyB, PIF6αΔC-nLUC, and cLUC-PIF6αΔC, the luciferase activity was retained in the dark, but it was undetectable under red light irradiation, whereas phyB^Q109A^ cotransfection with PIF6αΔC-nLUC and cLUC-PIF6αΔC had little effect on the luciferase activity (Fig. 5a). PhyB^Y276H^ can also inhibit the dimerization of PIF6αΔC (Fig. 5b). Moreover, structure prediction by AlphaFold 3 revealed that the PHY and PAS1 domains of full-length phyB occupy and interact with the bHLH domain of full-length PIF6α (Supplementary Fig. S20a). Pull-down assays revealed a weak interaction between phyB^Y276H^ and the bHLH domain of PIF6α (Supplementary Fig. S20b). These results suggest that phyB-Pfr interacts with both the N-terminus and the bHLH domain of PIF6, thereby disrupting PIF6 dimerization.

Next, we investigated the DNA-binding activity of bHLH domain-containing PIF6αΔC by the electrophoretic mobility shift assay (EMSA), using a reported G-box-containing probe^30^ and E-box-containing elements on the *FT* promoter^25^. The EMSA results revealed that PIF6αΔC exhibited robust DNA-binding activity, whereas with increasing concentrations of phyB under red light irradiation, the DNA-binding activity of PIF6αΔC was weakened, and parts of the DNA probes were retained in the gel well. In the dark, phyB remained in the Pr state, exhibiting little effect on the DNA-binding activity of PIF6αΔC (Fig. 5c). The addition of phyB^Y276H^ tended to result in retention of the DNA probes in the gel well (Supplementary Fig. S21). The phyB^Y276H/Q109A^ mutant hardly interacted with PIF6N (Supplementary Fig. S18) and had little influence on the DNA-binding activity of PIF6αΔC (Supplementary Fig. S21). Furthermore, we examined the transcriptional activation activity of PIF6 in mammalian cells by a transient dual-LUC assay^51^. PIF6αΔC activates the expression of the reporter gene, but this activation is inhibited when PIF6αΔC and phyB^Y276H^ are coexpressed in cells. In contrast, phyB^Y276H/Q109A^ barely inhibited the transcriptional activation activity of PIF6αΔC (Fig. 5d). These results suggest that phyB-Pfr influences DNA-binding and the transcriptional activation activities of PIF6.

## Discussion

The molecular mechanisms of Pr→Pfr photoconversion and PIF-mediated red light signal transduction of plant phytochromes remain poorly understood. On the basis of structural and biochemical analyses, we propose a model for the mechanisms underlying Pr→Pfr photoconversion and PIF6-mediated red light signal transduction of plant phyB (Fig. 5e). In phyB-Pr, two PSMs assemble in a “head-to-tail” manner and form a parallelogram-shaped platform together with two PAS2 domains. The HKRDs are located on this platform at a certain angle (Fig. 5e, left panel)^39^. Red light-irradiated phyB undergoes a series of conformational changes in multiple regions, including the PΦB-binding pocket, the HP loop, the PHY domain, the modulator loop, and helix 1 of the GAF domain (Fig. 3), and then it is converted to the Pfr form. The N-terminus of PIF6 stabilizes the “head-to-head” assembly of the two PSMs in phyB-Pfr. The structure of C-terminal region (PAS1-PAS2-HKRD domains) in phyB-Pfr requires further investigation (Fig. 5e, right panel). PhyB and PIF6 mutually regulate each other’s activities. Phytochrome A (phyA) is another important red/far-red light receptor in plants. PhyA can be activated by extremely low-intensity red light and can maintain its functions even under strong far-red light irradiation. Therefore, phyA allows seed germination and seedling de-etiolation under a thin layer of soil or in deep shade^16^. The PSMs of *Arabidopsis* phyA also adopt a “head-to-tail” assembly in the Pr state^52–54^, which is similar to the PSM assembly in phyB-Pr^39^. phyA-Pfr interacts with PIF1 and PIF3 to transduce red light signals^29^. Whether phyA possesses Pr→Pfr photoconversion and PIF-mediated signal transduction mechanisms similar to those of phyB requires further research.

During the preparation of our manuscript, Wang et al. also reported the complex structure of phyB-Pfr‒PIF6^43^. Although the structural aspects are similar, the focuses of the biochemistry experiments differ. Wang et al. primarily investigated the roles of phyB interacting with PIF6 in *Arabidopsis*. In contrast, in our study, we not only explored the photoactivation processes of phyB through biochemical approaches such as crosslinking and SAXS assays (Supplementary Fig. S15), but also investigated the effects of phyB-Pfr on the DNA-binding and transcriptional activation activities of PIF6 through EMSA, LCI, and dual-LUC (Fig. 5; Supplementary Fig. S21). Collectively, both studies reveal the photoactivation and signal transduction mechanisms of plant phyB and corroborate each other’s findings.

Phys widely exist in bacteria, fungi, algae, and plants. These organisms have evolved a large number of phytochrome proteins with different architectures. Canonical phys retain the conserved PAS-GAF-PHY architecture in the PSM but possess relatively diverse C-terminal regions for multiple functions^40,55^. Microorganism phytochromes generally do not contain PAS domains in their C-terminal regions^40,55^. The Pr→Pfr conversion mechanisms of bacterial phytochromes have been revealed^56,57^. Bacterial phytochromes adopt a “head-to-head” assembly mediated by the PAS-GAF domains in both the Pr and Pfr states. The PHY domains of bacterial phytochromes turn from the closed state to the open state during Pr→Pfr conversion (Supplementary Fig. S22)^56^. These observations are quite different from our findings concerning plant phyB in this study. The plant phys display a much more complicated architecture, containing two PAS domains (PAS1 and PAS2) in the C-terminal region (Supplementary Fig. S22), of which the PAS2 domain meditates a “head-to-tail” assembly of the PSMs in phyB-Pr. During Pr→Pfr photoconversion of the bacterial phys, the assembly of PSMs occurs consistently in a “head-to-head” manner, but in plant phys, the assembly of PSMs changes from “head-to-tail” to “head-to-head”. This conformational flexibility of plant phyB endows multiple functions and precise regulatory capacities in complex light environments.

The NTE region of phyB is flexible and invisible in the Pr state^39^, and it can act as an intrinsically disordered region (IDR) to modulate phyB phase separation and temperature sensing^22^. However, in the phyB‒PIF6β complex, the NTE region of phyB protomer A is stabilized by PIF6 and folds into three helices (Fig. 4a), implying that PIF6 is likely to regulate the phase separation and temperature sensing of phyB. According to the AlphaFold 3 prediction of the phyB‒PIF6α complex, the C-terminal region of phyB in the phyB‒PIF6α complex is close to the NTE region of phyB protomer B (Supplementary Fig. S20a), thereby inhibiting the binding of the second PIF6 molecule to phyB^Y276H^. In addition to interacting with PIFs, phyB-Pfr also interacts with multiple partners to transduce red light signals through its N-terminal PSM and/or C-terminal region^11^. In the phyB‒PIF6β complex, the PSM regions stabilized by PIF6 are resolved, but the C-terminal region comprising the PAS1, PAS2, and HKRD domains fails to be resolved due to the absence of a density map, implying that the proteins that interact with the C-terminal region of phyB-Pfr, including PHOTOPERIODIC CONTROL OF HYPOCOTYL1 (PCH1)^58^, might stabilize the conformation of this C-terminal region, thereby enabling the structural determination of the C-terminal region of phyB-Pfr.

Owing to the reversible interaction characteristics of phyB‒PIF (interaction under red light and dissociation under far-red light or in the dark), phyB and PIF6 have been applied in optogenetics to regulate a variety of biological processes^32–38^. In the phyB‒PIF6β complex, the N-terminus of PIF6β (residues 1‒60) has an extended structure and is sufficient to interact with phyB protomers A and B (Fig. 4a; Supplementary Figs. S16‒S19). These structural findings lay a foundation for the precise design of high-efficiency optical switches based on phyB and PIF6.

## Materials and methods

### Molecular cloning

The DNA sequences encoding the phyB (1‒1172, *At*2g18790) and PIF6α (1‒363, *At*3g62090) were amplified from the *Arabidopsis* cDNA library using a standard polymerase chain reaction (PCR)-based cloning strategy. *PhyB*, the constitutively active phyB mutant *PhyB*^*Y276H*^, and their mutants or truncations were subcloned to the pBAD vector (Invitrogen) which was modified with a N-terminal 3× Flag tag (MDYKDDDDKGDYKDDDDKIDYKDDDDK) for protein purification. *PIF6*α, *PIF6*α*ΔC*, and bHLH domain (S195‒P253) of PIF6α were subcloned into a modified pCold TF vector (Takara) with a large Trigger Factor (TF) tag at its N-terminus and a StrepII tag at their C-termini. The TF tag, generally considered non-disruptive to protein function, is a prokaryotic ribosome-associated chaperone protein (~48 kDa) that facilitates co-translational folding of newly expressed polypeptides^47,48^. PIF6β (1‒182) and the other PIFNs were subcloned to a modified pET15b vector (Novagen) with an N-terminus 6× His tag and a C-terminus StrepII tag (SAWSHPQFEKGGGSGGGSGGSAWSHPQFEK). The heme oxygenase gene (*HO1*) from *Synechocystis sp*. PCC 6803 and PΦB synthase enzyme gene (*HY2)* from *Arabidopsis* were subcloned into the pRSFDuet™-1 vector (Novagen), which is engineered to facilitate the simultaneous expression of two target open reading frames (ORF) with two separate T7*lac* promoter and ribosome binding site. The *Arabidopsis* phyB and its mutants were constructed by overlapping PCR. All constructs were generated using the Gibson assembly method and verified by sequencing.

### Protein expression and purification of phyB

The phyB‒PIF6β complex and their mutations or truncations were assembled by mixing the separately purified phyBs and PIF6β proteins. The wild-type phyB, phyB^Y276H^, phyBN (1‒908), phyBN^Y276H^ (1‒908), the PSMs domain (1‒624) of phyB and phyB^Y276H^, and relevant mutants were expressed in *E. coli* strain BL21(DE3) by co-expressing with the HO1 and HY2^41,44,45^. The transformed BL21 (DE3) was shaken and cultured in Lysogeny broth (LB) medium (10 mL, with 100 μg/mL ampicillin and 50 μg/mL kanamycin) at 37 °C overnight. The next day, the precultured *E. coli* (10 mL) was inoculated into one liter of terrific broth (TB) medium (supplemented with 100 μg/mL ampicillin and 50 μg/mL kanamycin) containing 0.4% glycerol and 1 mM MgCl_2_. When the cell density reached an OD_600_ of ∼1.0 to 1.2, the cultivation temperature was reduced to 16 °C for further induction. Subsequently, 1 mM isopropyl-β-d-thiogalactopyranoside (IPTG) was added to the medium followed by the addition of arabinose to 0.2% after an additional hour to induce PΦB and apoprotein synthesis.

The purification process for all phyB proteins was carried out at 4 °C. The bacterial pellet was collected by centrifugation, homogenized in ice-cold lysis buffer (25 mM Tris-HCl, pH 7.8, 150 mM NaCl, 5% glycerol, 1 mM phenylmethanesulfonyl-fluoride (PMSF)), and lysed using a high-pressure cell disrupter (JNBIO, China) at 1000 bar. Cell debris was removed by centrifugation at 20,000× *g* for 1 h at 4 °C, and the supernatant was loaded onto a column equipped with Ni^2+^ affinity resin (Ni-NTA, Qiagen). The column was washed with wash buffer (25 mM Tris-HCl, pH 7.8, 150 mM NaCl, 5% glycerol, 15 mM imidazole) and eluted with elution buffer (25 mM Tris-HCl, pH 7.8, 5% glycerol, 250 mM imidazole). The eluent was further loaded onto anti-FLAG G1 affinity resin (GenScript), washed with lysis buffer, and then eluted with lysis buffer supplemented with Flag peptide (GenScript). The eluted proteins were then subjected to a Source Q10/100 column (GE Healthcare), followed by a gradient NaCl elution (from 0 to 1 M) in 25 mM Tris-HCl, pH 7.8, 5% glycerol.

### Protein expression and purification of PIF6

PIF6α (1‒363), PIF6β (1‒182), PIF6αΔC (1‒247), and relevant mutants and truncations were expressed in the *E. coli* strain BL21(DE3). The transformed BL21 (DE3) was shaken and cultured in LB medium (10 mL, with 100 mg/mL ampicillin) at 37°C overnight. The next day, the precultured *E. coli* (10 mL) was inoculated into one liter of LB medium (supplemented with 100 mg/mL ampicillin). Then, one liter of mixture was shaken and cultured at 37 °C until the optical density at 600 nm reached 1.0. Then the culture was cooled to 16 °C and induced with 0.2 mM/L of IPTG. After 14‒16 h of growth at 16 °C, the bacterial pellet was collected and homogenized in 20 ml buffer A (25 mM Tris-HCl, pH 8.0, 150 mM NaCl). After high-pressure cell disruption and centrifugation at 20,000× *g* for 1 h at 4 °C, the supernatant was loaded onto a column equipped with 1 mL Ni^2+^ affinity resin twice. The resin bound with PIF6β was washed with 15 mL buffer B (25 mM Tris-HCl, pH 8.0, 150 mM NaCl, 15 mM imidazole), and eluted with 10 mL buffer C (25 mM Tris-HCl, pH 8.0, 250 mM imidazole). The resin bound with PIF6α or PIF6αΔC was washed with 15 mL buffer A (25 mM Tris-HCl, pH 8.0, 150 mM NaCl), and eluted with 10 mL buffer D (25 mM Tris-HCl, pH 8.0, 150 mM NaCl, 300 mM imidazole). The eluent of protein PIF6α or PIF6αΔC was incubated with Strep-Tactin Sepharose (IBA) at 4 °C for 2 h. The beads were washed five times with buffer E (100 mM Tris-HCl, pH 8.0, 150 mM NaCl, and 1 mM EDTA) and then eluted using an elution buffer containing 100 mM Tris-HCl, pH 8.0, 150 mM NaCl, 1 mM EDTA, and 2.5 mM d-desthiobiotin. The eluted proteins were then subjected to a Source Q10/100 column (GE Healthcare), followed by a gradient NaCl elution (up to 1 M) in 25 mM Tris-HCl, pH 8.0. The purity of the protein was examined using SDS-PAGE and visualized by Coomassie brilliant blue staining through all purification steps.

### Assembly of the phyB‒PIF6β complex

The phyB‒PIF6β complex and their mutations or truncations were assembled by mixing the separately purified phyBs and PIF6β proteins. To obtain the full-length protein samples for cryo-EM analysis, the purified phyB or phyB^Y276H^ and PIF6β were mixed at a molar ratio of ∼1:1.5 and incubated on ice under red light (665 nm, 800 μmol m^−2^ s^−1^) or in the dark for 20 min, respectively. Subsequently, the assembled phyB‒PIF6β or phyB^Y276H^‒PIF6β complex was purified on a Superose^TM^ 6 Increase 10/300 column (GE Healthcare) equilibrated with a buffer containing 25 mM HEPES, pH 7.8, 250 mM KCl, 10 mM DTT, 1 mM EDTA under red light (665 nm, 800 μmol m^−2^ s^−1^) or in the dark, respectively. To obtain the phyBN^Y276H^(M1‒S908) ‒PIF6β complex, the purified phyBN^Y276H^ protein was mixed with PIF6β protein at a molar ratio of ∼1:1.5 and further purified by size-exclusion chromatography on a Superose^TM^ 6 Increase 10/300 column equilibrated with a buffer containing 25 mM Tris-HCl, pH 8.0, 250 mM NaCl, 10 mM DTT, and 1 mM EDTA. The peak fractions were pooled and concentrated to approximately 2.0 mg mL^−1^ for further cryo-EM study.

### Cryo-EM sample preparation and data collection

For cryo-EM sample preparation, 20 μL of the purified phyB‒PIF6β complex was irradiated with red light (665 nm, 800 μmol m^−2^ s^−1^) for 20 min to promote the phyB stay in the Pfr state. The holey carbon grids (Quantifoil Au R1.2/1.3, 300 mesh or Quantifoil Cu R1.2/1.3, 300 mesh) were glow discharged at 20 mA for 120 s using a PELCO easiGlow glow discharge cleaning system (Ted Pella). Aliquots (3.5 μL) of freshly purified phyB‒PIF6β ( ~ 2 mg mL^−1^), phyB^Y276H^‒PIF6β ( ~ 2 mg mL^−1^), and the phyBN^Y276H^‒PIF6β complex ( ~ 0.7 mg mL^−1^) were applied onto the glow-discharged girds at 100% humidity and 8 °C. After blotting for 3.5 s by Whatman 597 filter paper with blot force value 0, the grids were plunged into liquid ethane cooled by liquid nitrogen in a Vitrobot (Mark IV; Thermo Fisher Scientific).

For cryo-EM data collection, the prepared grids were transferred to a 300 kV Titan Krios TEM (Thermo Fisher Scientific) equipped with a Gatan K3 detector and a GIF Quantum energy filter. For the phyB-Pfr‒PIF6β complex, images were recorded with a magnification of ×105,000, resulting in a final pixel size of 0.824 Å. Each image was recorded with 40 fractions using an electron beam with an expose rate of 15.938 e^‒^ pixel^‒1^ s^‒1^ for 2.14-s exposure with a final total electron dose of 50 e^‒^ Å^‒2^. Cryo-EM data for phyB^Y276H^‒PIF6β complex was collected by the same facility as that of the phyB-Pfr‒PIF6β complex, with some parameter modifications. The cryo-EM images were recorded with a magnification of ×105,000, resulting in a final pixel size of 0.84 Å. Each image was recorded with 40 fractions using an electron beam with an expose rate of 15.938 e^‒^ pixel^‒1^ s^‒1^ for 2.21-s exposure with a final total electron dose of 50 e^‒^ Å^‒2^. A summit direct electron detector with a slit width of 20 eV on the energy filter was used with a preset defocus range from ‒1.2 to ‒1.8 μm. All images were fully automated by the EPU software V3.2.0 and motion-corrected using MotionCor2 with a binning factor of 2 with dose weighting^55^. For the truncated phyBN^Y276H^ (M1‒S908) ‒PIF6β (M1‒A182) complex, images were recorded by another facility, with a magnification of 105,000×, resulting in a final pixel size of 0.85 Å. Each image was recorded with 40 fractions using an electron beam with an expose rate of 15.000 e^‒^ pixel^‒1^ s^‒1^ for 2.41-s exposure with a final total electron dose of 50 e^‒^ Å^‒2^. A summit direct electron detector with a slit width of 20 eV on the energy filter was used with a preset defocus range from ‒1.0 to ‒2.0 μm. The EPU software V.2.9 was used for fully automated data collection. All images were motion-corrected using MotionCor2 with a binning factor of 1 with dose weighting^59^.

### Cryo-EM data processing, model building, and refinement

The diagrams of the procedures for data processing of phyB‒PIF6β, phyB^Y276H^‒PIF6β, and phyBN^Y276H^(M1‒S908)‒PIF6β complex are presented in Supplementary Figs. S2, S8, and S11, respectively. For the phyB-Pfr‒PIF6β complex, a total of 5769 movies were collected. Frames of individual movies were aligned with MotionCor2 and the defocus values were estimated with Patch CTF in cryoSPARC v4.5^59–61^. Subsequently, 5767 micrographs were selected for further processing, which is primarily done in cryoSPARC v4.5 unless specified. 9,028,696 particles were picked from motion-corrected images with Blob Picker and extracted with a box size of 280 pixels with Particle Extraction. The resulting particles were first sorted by 2D classification (*N* = 100) and followed by Ab Initio Reconstruction (*N* = 10), in which four classes containing 2,776,979 particles The best class containing a total of 1,160,077 particles were classified using 3D Class, resulting in 12 different classes. The class with the best features was refined with Non-uniform refinement, resulting in consensus maps with a global resolution of 3.08 Å, which is estimated by the 3D FSC^62^. In addition, the local resolution of these maps ranged from 2.5 to 5.0 Å, which is estimated by Monores^63^.

For the phyB^Y276H^‒PIF6β complex, a total of 6243 movies were collected. Frames of individual movies were aligned with MotionCor2, and the defocus values were estimated with Patch CTF in cryoSPARC v4.5. Subsequently, 6160 micrographs were selected for further processing, which is primarily done in cryoSPARC v4.5 unless specified. 4,564,364 particles were picked from motion-corrected images with Blob Picker and extracted with a box size of 280 pixels with Particle Extraction. The resulting particles were first sorted by 2D classification (*N* = 100) and followed by Ab Initio Reconstruction (*N* = 10), in which 3 classes contained 2,027,852 particles. The best classes were classified using 3D Class, resulting in 10 different classes. The class with the best features, containing 661,628 particles, was refined with Non-uniform refinement, resulting in consensus maps with a global resolution of 2.60 Å, which is estimated by the 3D FSC^62^. To address the preferred orientation problem, a 30°-tilt data collection strategy was used. In detail, a total of 2049 movies were collected and 1932 movies were selected for further processing. Finally, a total of 2,989,142 particles were merged into the particles (from the non-tilted data set as described before) to perform 3D classification, resulting in 12 classes. The best class was chosen to be further refined by Non-uniform refinement, resulting in consensus maps with a global resolution of 3.10 Å, which is estimated by the 3D FSC^62^. In addition, the local resolution of these maps ranged from 3.0 to 6.0 Å, which is estimated by Monores^63^.

For the phyBN^Y276H^‒PIF6β complex, a total of 5401 movies were collected. Frames of individual movies were aligned with MotionCor2 and the defocus values were estimated with Patch CTF in cryoSPARC v4.5. Subsequently, 5327 micrographs were selected for further processing, which is primarily done in cryoSPARC v4.5 unless specified. 3,054,469 particles were picked from motion-corrected images with Blob Picker and extracted with a box size of 280 pixels with Particle Extraction. The resulting particles were first sorted by 2D classification (*N* = 100) and followed by Ab Initio Reconstruction (*N* = 10), in which three classes contained 2,063,526 particles. The best classes were classified using 3D Class, resulting in 10 different classes. The class with the best features, containing 565,463 particles, was refined with Non-uniform refinement, resulting in consensus maps with a global resolution of 2.87 Å, which is estimated by the 3D FSC^62^. In addition, the local resolution of these maps ranged from 2.5 to 5.0 Å, which is estimated by Monores^63^.

The initial coordinate files for the three structures were obtained through Relion-5.0 ModelAngelo^64^. The initial model was manually adjusted using COOT^65^, resulting in an optimized model. The complex model was refined in real space using PHENIX, with secondary structure and geometric restraints applied^66^. The model quality was assessed using MolProbity scores^67^, Ramachandran plots, and EMRinger^68^. Figures were generated using ChimeraX v.1.2.5 and PyMol v.2.5.1.

### Thiol-directed chemical crosslinking assay

To perform the thiol-directed chemical crosslinking assays, we introduced a cysteine mutation T449C for specific crosslinking. We also introduced C925S, C936S, C972S, and C1121S into phyB^T449C^ and phyB^Y276H/T449C^ to avoid nonspecific crosslinking. The proteins phyB^T449C^, phyB^Y276H/T449C^, and PIF6 N-terminal fragment (PIF6N, residues 13‒100) were prepared in a buffer containing 25 mM Tris-HCl, pH 8.0, 150 mM NaCl. To maintain all phyB^T449C^ proteins in the Pr or Pfr forms, 50 μL of purified proteins were irradiated with a far-red light (730 nm, 1156 μmol m^−2^ s^−1^) or red light (665 nm, 767 μmol m^−2^ s^−1^) for 20 min, respectively. The crosslinker 1,2-ethanediyl bismethanethiosulfonate (M2M) was prepared at a concentration of 100 mM in DMSO. M2M was mixed with phyB^T449C^, phyB^Y276H/T449C^, the phyB^T449C^ + PIF6N protein mixture, and the phyB^Y276H/T449C^ + PIF6N protein mixture at a molar ratio of 500:1, respectively. Approximately 2 μM protein was incubated with 1 mM M2M on ice for 30 min. The assay was conducted under far-red light (730 nm, 1156 μmol m^−2^ s^−1^) or red light (665 nm, 767 μmol m^−2^ s^−1^) from the beginning to the end. The reaction mixture was analyzed by SDS-PAGE and Coomassie blue staining in the presence or absence of 100 mM DTT. The experiments were repeated four times.

### Immunoblot assays

To verify the interactions between phyB or phyB^Y276H^ and bHLH domain (S195‒P253) of PIF6α, bHLH was fused with a StrepII tag, and phyB or phyB^Y276H^ was fused with a 3× Flag tag. All proteins were expressed in the *E. coli* strain BL21 (DE3). The bHLH was mixed with phyB or phyB^Y276H^ and BSA in buffer E (100 mM Tris-HCl, pH 8.0, 150 mM NaCl, and 1 mM EDTA) and incubated with Strep-Tactin Sepharose under red light (665 nm, 800 μmol m^−2^ s^−1^) or in the dark at 4 °C for 2 h, respectively. The bHLH was also mixed with phyB or phyB^Y276H^ and BSA in buffer F (50 mM Tris-HCl, pH 7.8, 150 mM NaCl, 5% glycerol) and incubated with anti-FLAG G1 affinity resin under red light (665 nm, 800 μmol m^−2^ s^−1^) or in the dark at 4 °C for 2 h, respectively. The beads were washed five times with buffer E or buffer F, and then eluted with buffer E supplemented with 2.5 mM d-desthiobiotin or buffer F supplemented with 0.3 mg/mL Flag peptide, respectively. Proteins in the input and elution were detected by immunoblots probed with antibodies against StrepII (Abbkine, Cat Number: ABT2230) and Flag (Abbkine, Cat Number: ABT2010) (all antibodies were used at 1:3000 dilution). This experiment was independently repeated three times.

### SEC assays

All proteins used in the SEC assays were expressed in *E. coli* strain BL21(DE3) as described above. The proteins were run on a Superose^TM^ 6 Increase 10/300 column (GE Healthcare) equilibrated with buffer (25 mM Tris-HCl, pH 8.0, 150 mM NaCl, 5 mM DTT). The elution peak of the protein was collected in individual tubes (500 μL per tube). Samples from relevant fractions were analyzed by SDS-PAGE and visualized using Coomassie blue staining.

### In vitro pull-down assays

To verify the interaction between truncated PIF6 and phyB, PIF6α, PIF6β, and PIF6αΔC were fused with a StrepII tag, phyB, and phyB^Y276H^ were fused with a 3× Flag tag. All proteins were expressed in the *E. coli* strain BL21 (DE3). The PIF6α, PIF6β, and PIF6αΔC proteins were mixed with phyB^Y276H^ in buffer E (100 mM Tris-HCl, pH 8.0, 150 mM NaCl, and 1 mM EDTA) and incubated with Strep-Tactin Sepharose (IBA) in the dark at 4 °C for 2 h. The PIF6α, PIF6β, and PIF6αΔC proteins were mixed with phyB in buffer E and incubated with Strep-Tactin Sepharose under red light (665 nm, 800 μmol m^−2^ s^−1^) or in the dark at 4 °C for 2 h. The beads were washed five times with buffer E and then eluted with buffer E supplemented with 2.5 mM d-desthiobiotin. The input and eluent were analyzed by 12% SDS-PAGE and either stained for protein with Coomassie blue or for the covalently bound PΦB by zinc-induced fluorescence.

To verify the interaction between PIFNs and phyB, all PIFNs are fused with a StrepII tag, while phyB or phyB^Y276H^ are fused with a 3× Flag tag. The pull-down experiment was conducted according to the steps described previously. The input and eluent were analyzed by 12% SDS-PAGE and visualized using Coomassie blue staining. To verify the interaction of truncated phyB or phyB^Y276H^ and PIF6β, PIF6β was fused with a StrepII tag, truncated phyB or phyB^Y276H^ was fused with Flag tag, and HKRD was fused with His tag. All proteins were obtained in *E. coli* strain BL21 (DE3) and were purified on a Superose^TM^ 6 Increase 10/300 SEC column equilibrated with the buffer containing 25 mM Tris-HCl, pH 8.0, 150 mM NaCl, and 5 mM DTT. Conduct the pull-down experiment according to the steps described previously.

To identify key residues that mediate the phyBN^Y276H^‒PIF6N or phyBN‒PIF6N interaction, StrepII-tagged PIF6N (1‒64) was used to pull down Flag-tagged phyBN or phyBN^Y276H^ and their site-directed mutants. Various truncations and site-directed mutants of phyB and PIF6 were expressed in *E. coli* strain BL21 (DE3). The pull-down experiment was conducted according to the steps described previously. The input and eluent were analyzed by 12% SDS-PAGE and either stained for protein with Coomassie blue or for the covalently bound PΦB by zinc-induced fluorescence.

### EMSA

For EMSA assay, the 6-Carboxyfluorescein (FAM)-labeled oligonucleotides (G-wt: GGCCGAGGTGAGTAGGACACGTGGACACGTCTTCCGAA^30^, FT-E Box1: AAGAAGAGAAATAAAACAATTGATTTGGTTTATATTAT, FT-E Box2: AATAGGTGACTATTCTCAAATGTCCTGGTTCTATCTAA, FT-E Box3: AAAAATTAGTGGCTACCAAGTGGGAGATATAATTTGGA, and FT-E Box4: ATCAACACAGAGAAACCACCTGTTTGTTCAAGATCAAA) were synthesized (Tianyi Biotech)^25^, dissolved in ultrapure water and annealed by incubating in boiling water, followed by gradual cooling to room temperature. To investigate the interaction between G-wt or FT-E Boxes and PIF6ΔC, G-wt (15 nM) was incubated with 0.375, 0.75, 1.5, and 3.0 µM PIF6ΔC proteins on ice for 90 min in EMSA buffer (20 mM HEPES, pH 7.8, 75 mM KCl, 5 mM MgCl_2_, 5 mM DTT, 0.5 mg mL^−1^ BSA, and 10% glycerol) with 200 ng ml^−1^ heparin, respectively.

To examine the impact of phyB^Y276H^ on the binding of PIF6ΔC to G-wt, PIF6ΔC (3 µM) and G-wt or FT-E Boxes (15 nM) were incubated with 0.75, 1.5, 3.0, 4.5, and 6.0 µM phyB^Y276H^ protein on ice for 90 min in EMSA buffer with 200 ng mL^−1^ heparin, respectively. The reactions were resolved on 6% native acrylamide gels (37.5:1 acrylamide: bis-acrylamide). Images of the gels were obtained using Amersham Typhoon (GE Healthcare). To examine the impact of phyB on the binding of PIF6ΔC to G-wt, PIF6ΔC (1.5 µM) were incubated with 0.75, 1.5, 3.0, 4.5, and 6.0 µM phyB protein on ice under red light (665 nm, 800 μmol m^−2^ s^−1^) or in the dark for 1 h. Then, the mixture and G-wt (15 nM) were incubated in EMSA buffer with 200 ng mL^−1^ heparin on ice under red light (665 nm, 800 μmol m^−2^ s^−1^) or in the dark for 1 h, respectively. The reactions were resolved on 6% native acrylamide gels (37.5:1 acrylamide:bis-acrylamide) under red light (665 nm, 800 μmol m^−2^ s^−1^) or in the dark for 30 min, respectively. Images of the gels were obtained using Amersham Typhoon (GE Healthcare).

### SAXS measurements

For SAXS experiments, the sample preparation of phyB‒PIF6β was similar to that of Cryo-EM sample preparation, and all proteins were purified using a Superose^TM^ 6 Increase 10/300 SEC column equilibrated with a buffer containing 25 mM HEPES, pH 7.8, 250 mM KCl, 10 mM DTT, 1 mM EDTA. To accumulate phyB in Pfr form, 90 μl phyB protein (18 μM) and phyB‒PIF6β complex (18 μM) were first irradiated with red light (665 nm, 800 μmol m^−2^ s ^−1^) at room temperature for 15 min. To maintain phyB in Pr form, 90 μL purified proteins were irradiated with a far-red light (730 nm, 1156 μmol m^−2^ s ^−1^) laser at room temperature for 15 min. SAXS data for the protein solutions of phyB-Pr was collected under dark conditions. SAXS data for phyB-Pfr and phyB‒PIF6β were collected under red light (665 nm, ~600 μmol m^−2^ s^−1^). All SAXS data for the protein solutions were collected at the SSRF using the BL19U2 beamline at room temperature. For each measurement, 20 consecutive frames with 1 s exposure time were recorded, averaged after checking that there was no difference between the first and last frames of the SAXS data, and processed using the RAW software (bioxtasraw.sourceforge.net). Similarly, the background data were recorded using a sample buffer and were subtracted from the protein patterns.

### Dual luciferase assay

The ‒1034 to ‒1 fragment of the *FLOWERING LOCUS T* (FT) promoter was inserted into the modified PNL2.2 vector (Promega, N1071)^51^ to drive the expression of luciferase. HeLa cells were cultured in Dulbecco’s modified Eagle medium (DMEM) (Gibco) supplemented with 10% FBS, 100 U/mL penicillin, and 100 µg/mL streptomycin. The cells transiently cotransfected with ~0.2 μg of PIF6^1−247^, pNL2.2 *pFT::Luc SV40::Ren*, ~2 μg of phyB^Y276H^-NLS and 7.2 μg 40 kDa linear polyethylenimine (PEI) (Polysciences) when cell density reached 70%‒80%. The transfected cells were cultured for 48 h before harvesting. The experiment was performed according to the manufacture’s manual for the Dual Luciferase Reporter Gene Assay Kit (Vazyme, Cat Number: DL101-01). This experiment was independently repeated three times. For the phyB combination, the transfection conditions were similar to the description above, and were irradiated with far-red light (730 nm, 654 μmol m^−2^ s ^−1^) and red light (665 nm, 866 μmol m^−2^ s ^−1^) for 20 h before harvested.

### LCI assay

The coding sequences of PIF6αΔC and phyB/phyB^Y276H^/phyB^Q109A^ were cloned into pCAMBIA1300-Nluc/Cluc and pPlink, respectively. The constructs were transformed into *Agrobacterium tumefaciens* GV3101 using the freeze-thaw method^69^. Overnight cultures were suspended in an infiltration solution (10 mM MES, pH 5.6 and 10 mM MgCl_2_ containing 200 μM acetosyringone). The solution was then infiltrated into *N. benthamiana* leaves. After 2 d, 1 mM fluorescein potassium salt was sprayed onto the abaxial surfaces of the leaves, and photographs were taken using a NightSHADE LB 985 imaging system. The combination of phyB and phyB^Q109A^ was irradiated with a far-red light laser (730 nm, 654 μmol m^−2^ s ^−1^) and red light laser (665 nm, 866 μmol m^−2^ s ^−1^) for 5 min before imaging.

### UV–vis spectroscopy and kinetic analyses

UV–vis absorption spectra for the Pr-Pfr photoconversion of phyB, phyB^Y276H^, phyB + PIF6β, and phyB + PIF6β^3 mut^ were measured at 25 °C by Cary60 spectrophotometer (Agilent) using red light (665 nm) and far red light (730 nm) to drive Pr→Pfr and Pfr→Pr photoconversion, respectively. In order to maintain consistency among all measurements, experiments were performed using the same cuvette and in the same sample volume. For all photoconversion reactions, the sample concentration was adjusted so that the maximum absorption of the Q band in the Pr state was ~0.4. To maintain the consistency of spectral measurements, all proteins for the assays were purified using a Superose^TM^ 6 Increase 10/300 column equilibrated with buffer containing 25 mM HEPES, pH 7.8, 250 mM KCl, 10 mM DTT, 1 mM EDTA. PIF6β was incubated with phyB at the same concentration ( ~1.0 mg/mL) for full-wavelength spectral scanning. For the measurements of Pr→Pfr photoconversion, phyB samples were irradiated with far-red light (730 nm, 655 μmol m^−2^ s ^−1^) for ~10 min to ensure all phyB proteins remained in the Pr state. Pr spectra were collected from 300 to 900 nm as soon as possible (within 1.5 s). Then the samples were exposed to red light (665 nm, 867 μmol m^−2^ s^−1^) until they reached steady state, after which Pfr spectra were collected as described above. The absorbance at 665 nm and 720 nm was recorded. Time-resolved spectra were collected from 300 to 900 nm in 5-nm intervals, with a scan rate set at 24,000 nm/min. SCRs were calculated from the Pr-minus-Pfr absorbance difference spectra (Δabsorbance of the Q band maximum in the Pr state/Δabsorption of the Q-band minimum in the Pfr state).

For assays in Fig. 4d, e, photoconversion was measured at 25 °C under red light (665 nm, 617 μmol m^−2^ s^−1^) or far-red light (730 nm, 655 μmol·m^−2^·s^−1^). Pfr→Pr thermal reversion in Fig. 4f was measured at 25 °C in the dark after driving the Pfr levels to steady state with red light (665 nm, 867 μmol m^−2^ s^−1^). The absorbance at 665 nm or 720 nm was recorded with corresponding time intervals to evaluate photoconversion and thermal reversion of phyB, phyB + PIF6β, and phyB + PIF6β^3 mut^. The kinetic rate constants for phyB photoconversion and thermal reversion were calculated using the equation: Abs_i_ = ΔAbs_i_ ⋅exp(-k⋅t) + Abs (∞), where i represents the *i*th wavelength, Abs is the absorption, ΔAbs is the reaction amplitude, *k* is the rate constant, *t* is time, and Abs (∞) is the absorption at time zero. If two exponentials were required to explain the results, the data were fit to a simple sum of two exponentials using the equation described above^70–72^. All spectroscopy data were collected using Cary WinUV software. All data were processed and fitted using GraphPad Prism9.5 and Origin 2024.

## Supplementary information

Supplementary information

Supplementary movie1

## Data Availability

Atomic coordinates and structure factors of full-length phyB-Pfr‒PIF6β complex, phyB^Y276H^‒PIF6β complex, and phyBN^Y276H^ (1‒908) ‒PIF6β complex have been deposited in the PDB under accession codes 9JLB, 9ITF, and 9IRK, respectively. Accession codes of cryo-EM maps for full-length phyB-Pfr‒PIF6β complex, phyB^Y276H^‒PIF6β complex, and phyBN^Y276H^ (1‒908)‒PIF6β complex in the EM database are EMD-61582, EMD-60860, and EMD-60816, respectively. All the other data are presented in the main text and in the Supplementary Materials.
