# Supplementary material for: Structural insight into PIF6-mediated red light signal transduction of plant phytochrome B
Source: Cell Discov. 2025 May 22;11:51. doi: 10.1038/s41421-025-00802-3 (PMC12098889; doi:10.1038/s41421-025-00802-3)
Supplement: Supplementary file 1 — Supplementary information [file 41421_2025_802_MOESM1_ESM.pdf]

Structural insight into PIF6-mediated red light signal transduction of plant phytochrome B

Hanli Jia[1]†, Zeyuan Guan[1]†, Junya Ding[1]†, Xiaoyu Wang[1]†, Dingfang Tian[1], Yan Zhu[1], Delin Zhang[1], Zhu Liu[1], Ling Ma[1]*, and Ping Yin[1]*

[1]National Key Laboratory of Crop Genetic Improvement, Hubei Hongshan Laboratory, Huazhong Agricultural University, Wuhan 430070, China.

†These authors contributed equally to this work.

*To whom correspondence should be addressed:

Ping Yin, E-mail: yinping@mail.hzau.edu.cn

Ling Ma, E-mail: lingma@mail.hzau.edu.cn

This file includes:

Supplementary Figures 1–22

Supplementary Tables 1–3

Supplementary Movie 1

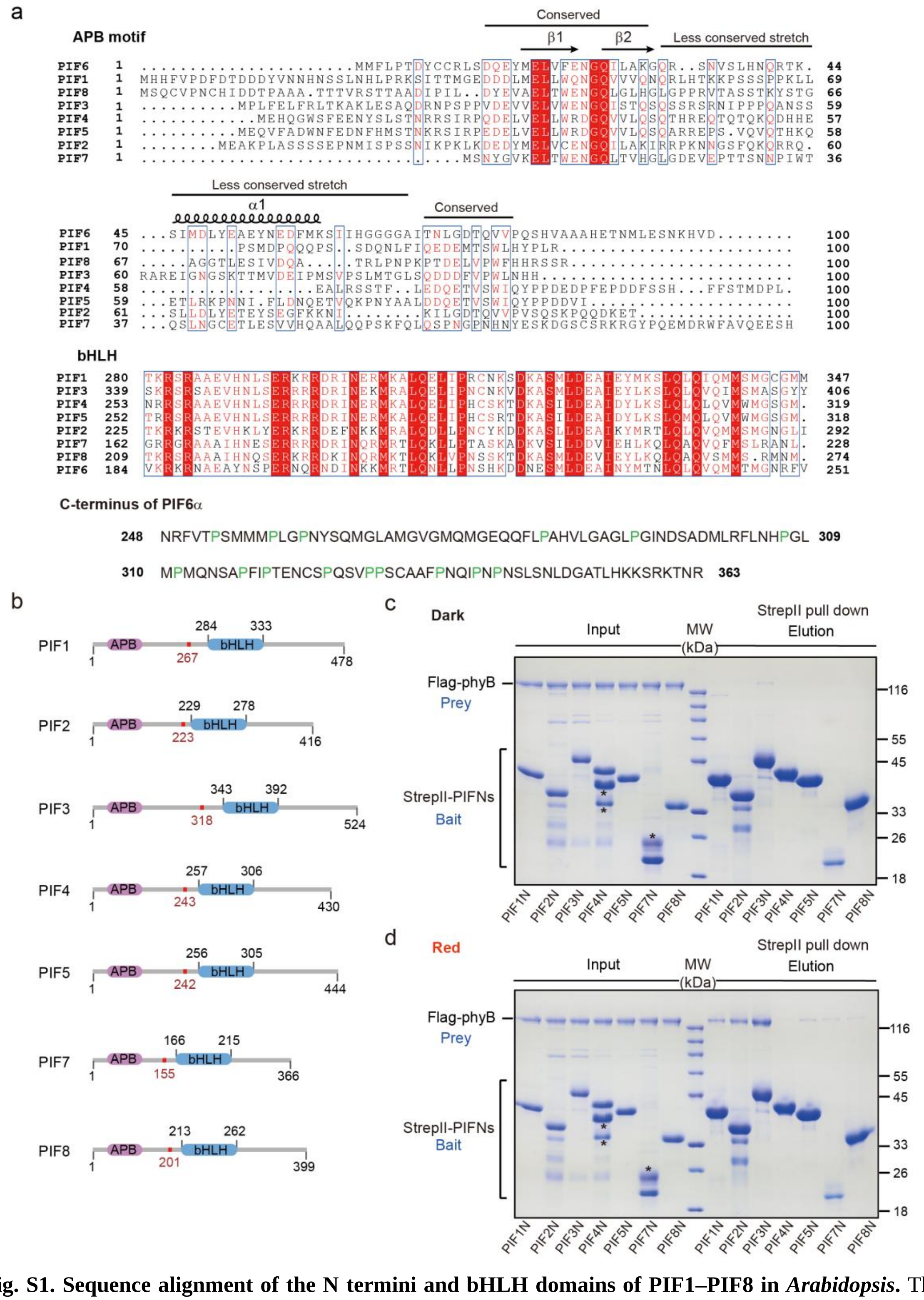

Fig. S1. Sequence alignment of the N termini and bHLH domains of PIF1–PIF8 in *Arabidopsis*. The alignment is performed using the MultAlin and ENDscript programs (http://multalin.toulouse.inra.fr/multalin/multalin.html). Sequence identity is indicated by white letters against a red background, and sequences with similarity over 90% are shown in red letters. Numbers of amino acids are marked at both ends of the sequence. (**a**) Sequence alignment of the N-terminuses and the bHLH

domains of *Arabidopsis* PIF1–PIF8. APB: active phyB-binding motif. bHLH: basic helix-loop-helix. C-terminus of PIF6α is shown. (**b**) Domain organizations of *Arabidopsis* PIF1–5 and PIF7–8. The active phyB-binding motif (APB) and basic helix-loop-helix (bHLH) domain are shown. The numbers denote the location of domain features within the PIFs. Red numbers indicate the expressing boundaries of different PIFs (PIFNs) described in (c) and (d). The interactions between phyB and PIFNs are assessed by pull-down assays in the dark (**c**) and under red light irradiation (**d**). All PIFNs are fused with a StrepII tag, while phyB is fused with 3×Flag tag. Protein mixtures containing phyB and different PIFNs in the "Input" are incubated with Strep-Tactin Sepharose under red light (800 μmol m^{-2}s^{-1}) or in the dark for 2 h.

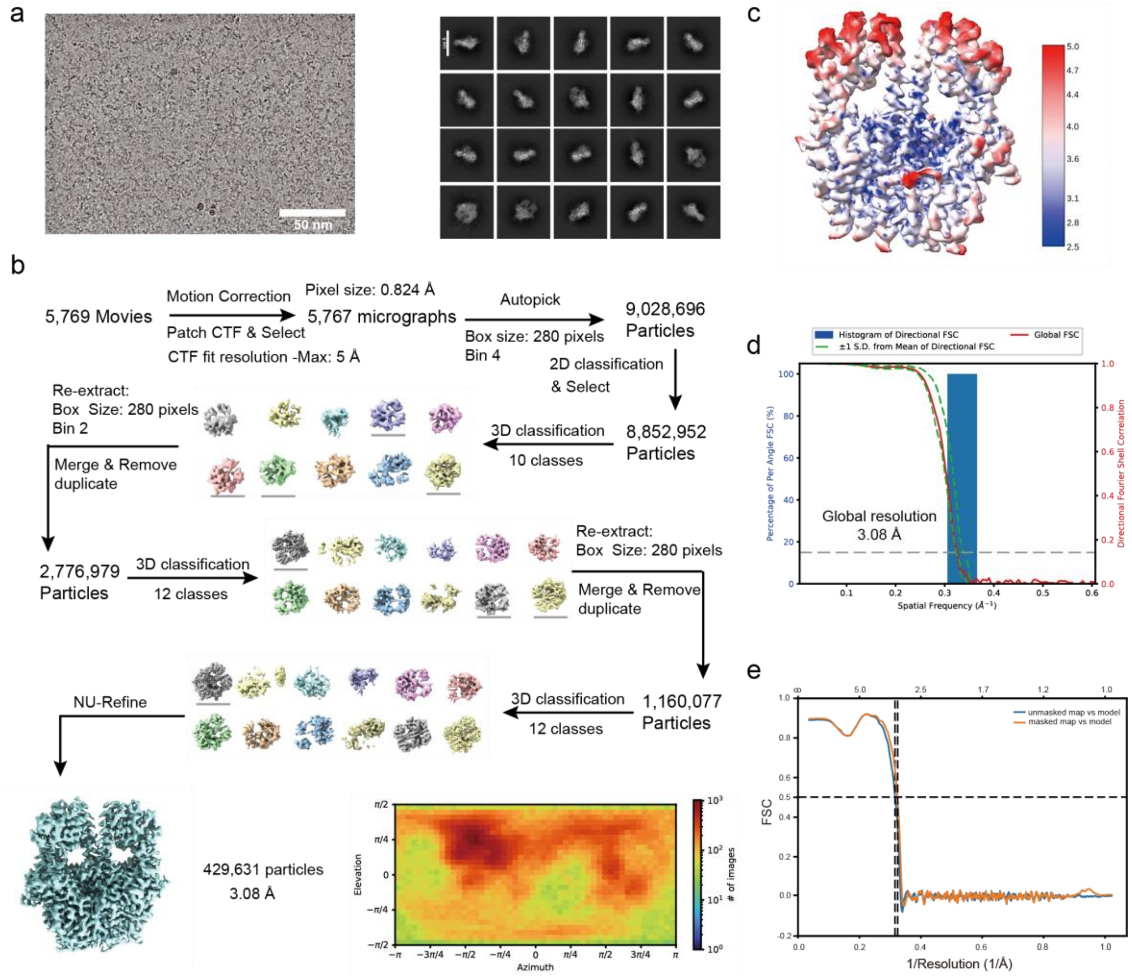

Fig. S2. Cryo-EM data processing of the phyB-Pfr–PIF6β complex. (**a**) Representative micrograph and 2D class averages of the phyB-Pfr–PIF6β complex. (**b**) Flowchart for the cryo-EM data processing of the phyB-Pfr–PIF6β complex and orientation distribution plot for the final reconstruction. (**c**) Local resolution (right panel) from 3D refinement for the final reconstructions. (**d**) 3D FSC curves of 3D reconstruction. (**e**) Refinement and map-versus-model FSC.

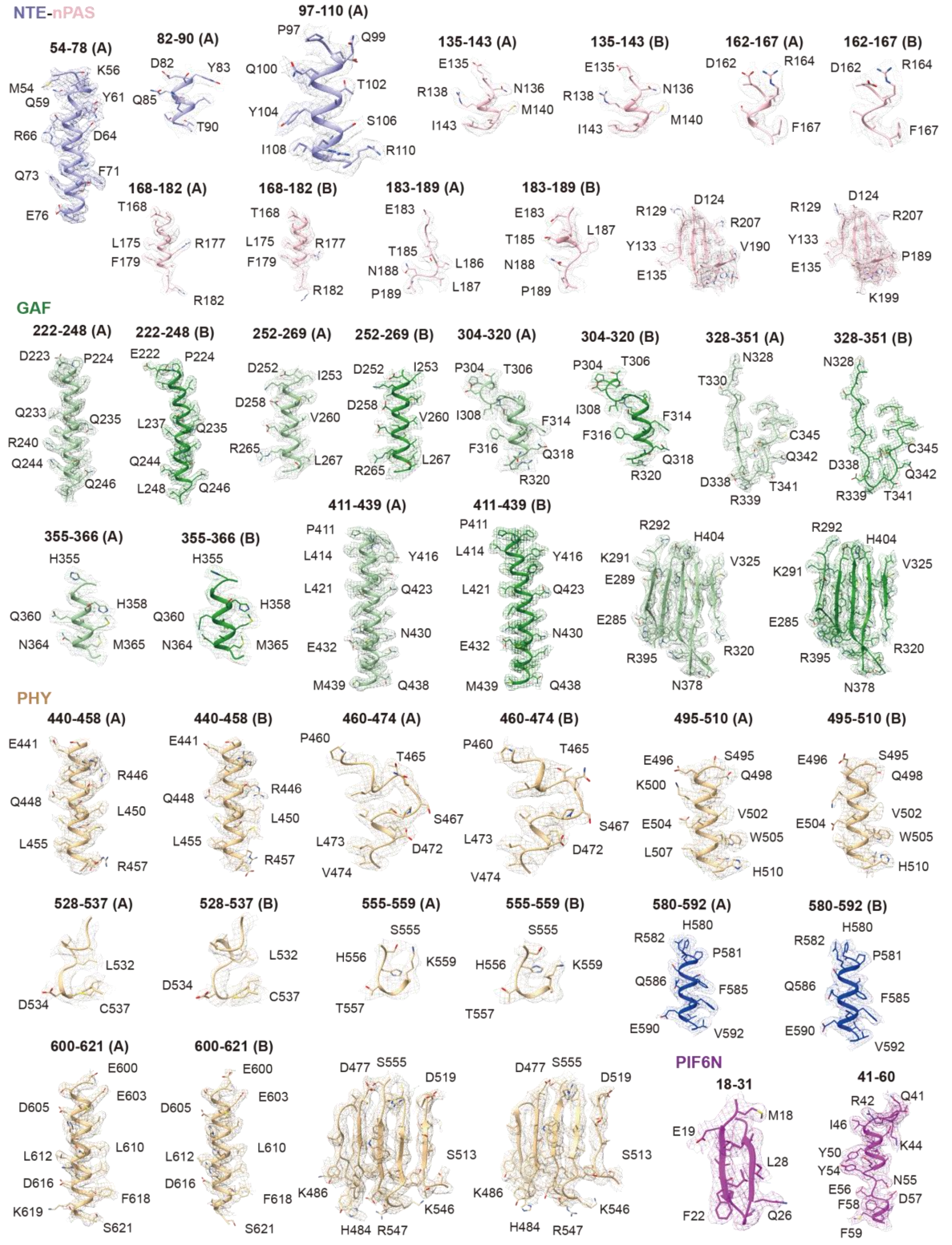

Fig. S3. Representative Cryo-EM density maps for key components of the phyB-Pfr–PIF6β complex. Domains in protomer A are labelled with "(A)"; domains in protomer B are labelled with "(B)". The selected bulky residues are labelled for each segment. The densities shown in this figure are generated in ChimeraX, with the map (EMD- 61582).

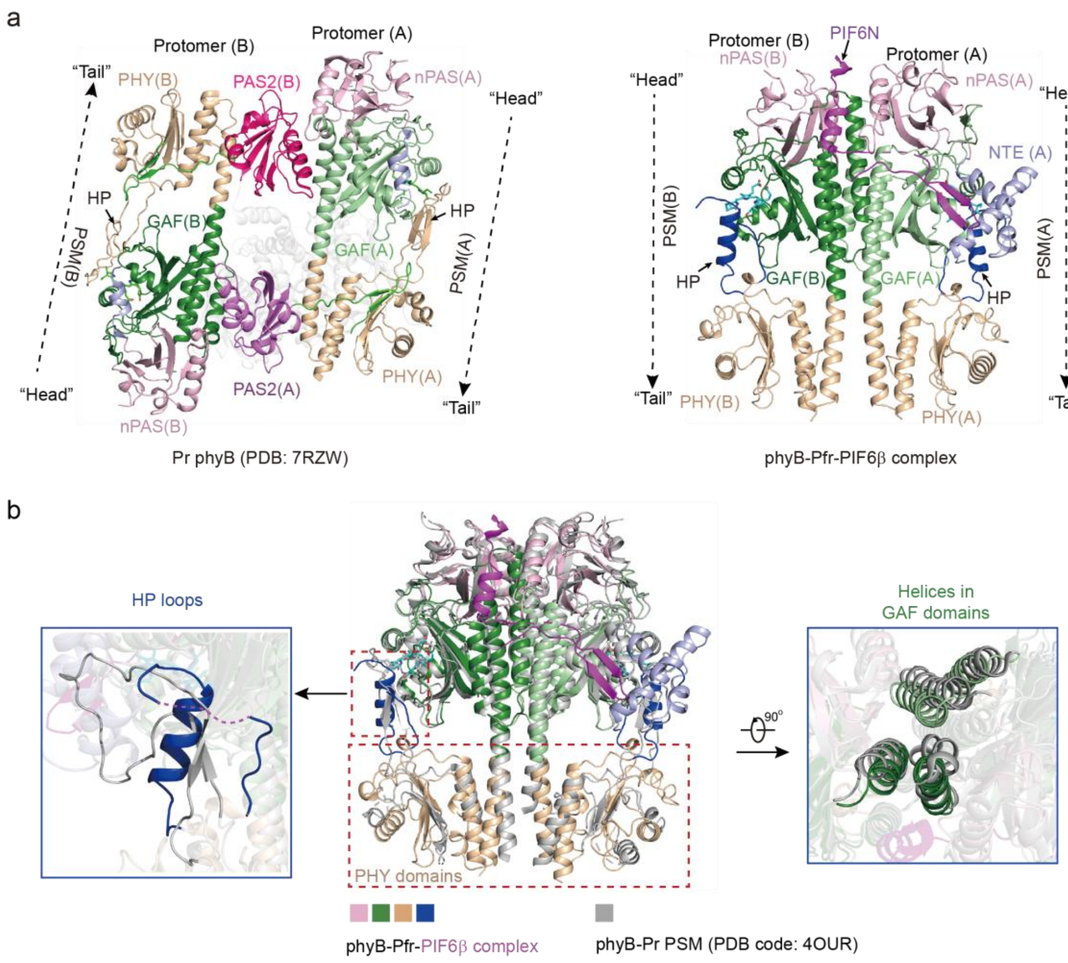

Fig. S4. Structural comparison of phyB-Pr and PIF6β-bound phyB-Pfr. (**a**) Left panel shows the structure of full-length phyB in the Pr form (PDB code: 7RZW). The photosensory module (PSM) of phyB comprises the NTE, nPAS domain, GAF domain, and PHY domain. Two PSMs and two PAS2 domains form a parallelogram-shaped platform, and two PSMs assembles in a "head-to-tail" manner under the mediation of the PAS2 domain. The HP loop folds into a pair of β-sheets. Right panel shows the structure of phyB-Pfr–PIF6β complex in this study. Two PSMs assemble in a "head-to-head" manner. The HP loop folds into an α-helix. NTE, N-terminal extension; nPAS, N-terminal Period/Arnt/Sim domain; GAF, cGMP phosphodiesterase/adenylyl cyclase/FhlA domain; PHY, phytochrome-specific domain; HP, hairpin loop. Domains in protomer A are labelled with "(A)"; domains in protomer B are labelled with "(B)". (**b**) Structures comparison of phyB-Pr PSM (PDB code: 4OUR)[41] and PIF6β-bound phyB-Pfr dimer. A series of conformational changes exist in the PHY domain, the HP loop, and the GAF domain.

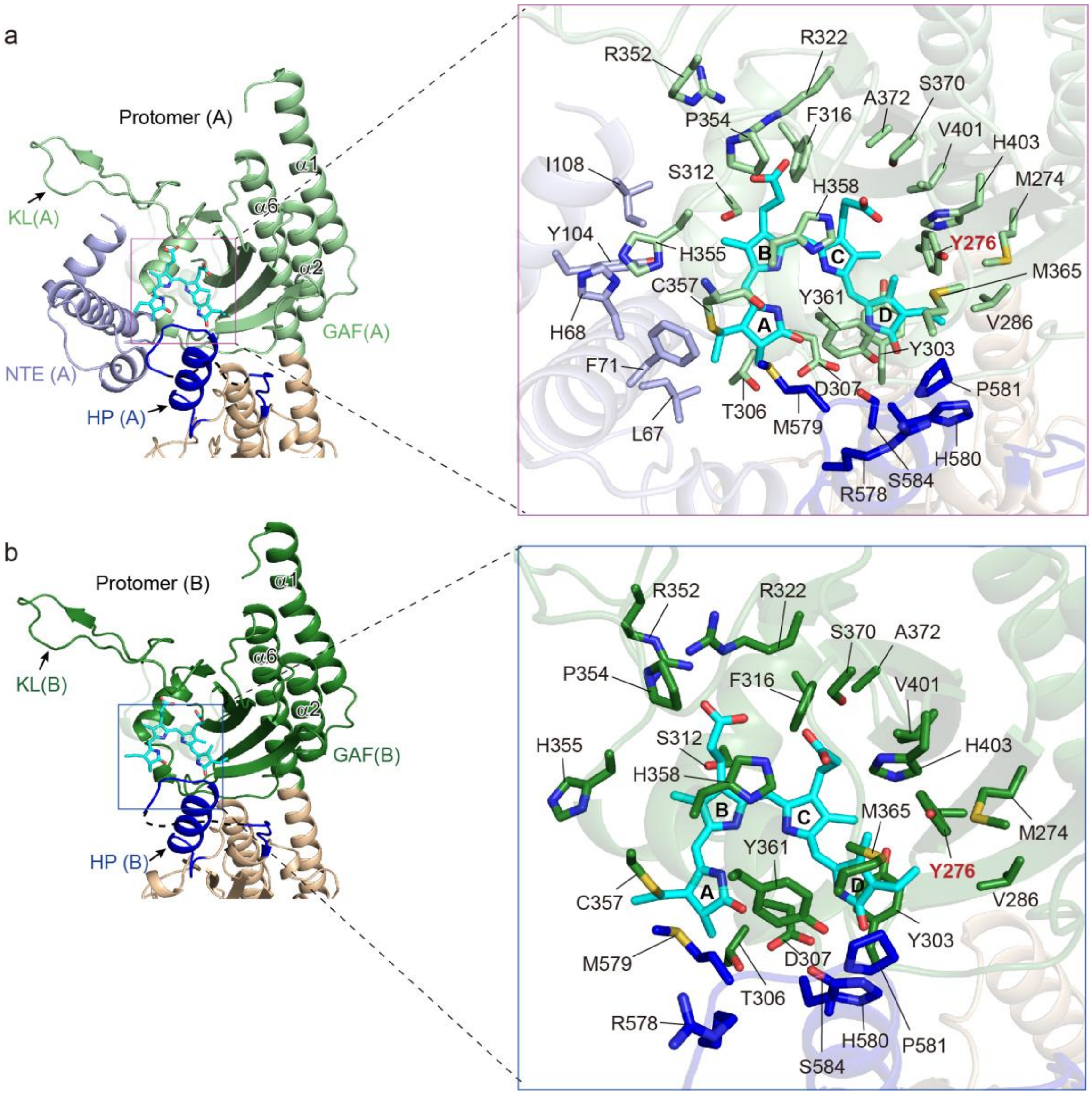

Fig. S5. PΦB binding pockets of the phyB-Pfr–PIF6β complex. The PΦB binding pocket of phyB-Pfr protomer A (**a**) and phyB-Pfr protomer B (**b**). The side chains of the amino acids adjacent to the PΦB are depicted as sticks. Residues from the NTE, GAF, and hairpin loops are colored light blue, light or dark green, and blue, respectively. PΦB is shown in cyan and is covalently bound by C357. PΦB is in an "*E*" configuration. The NTE in PIF6β-bound phyB-Pfr protomer A folds into three helices, while the NTE in phyB-Pfr protomer B is invisible. HP loop folds into an α-helix. NTE, N-terminal extension; GAF, cGMP phosphodiesterase/adenylyl cyclase/FhlA domain; HP, hairpin loop; KL, knot lasso in GAF domain.

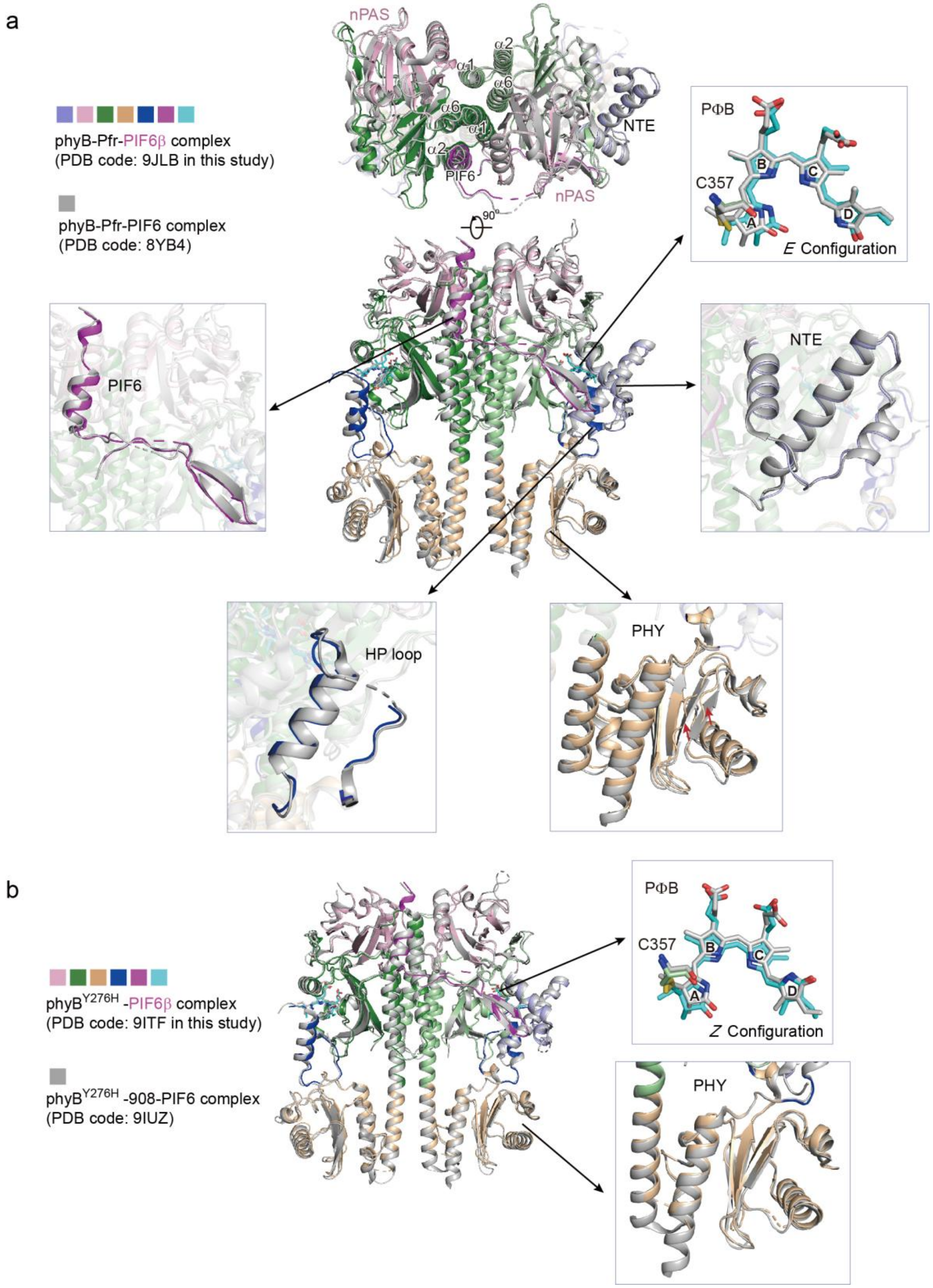

Fig. S6. Structural superposition of the phyB-Pfr–PIF6 complexes in this study and in Wang et al.[43]. (**a**) Structural comparison of chains A and B in phyB-Pfr–PIF6β complex (PDB code: 9JLB in this study) and chains B and C in phyB-Pfr–PIF6 complex (PDB code: 8YB4 in Wang et al.[43]). The overall folds of

these two structures are nearly identical. The β strands in the PHY domain (indicated by red arrows) slightly differ. NTE, N-terminal extension; HP, hairpin loop; PHY, phytochrome-specific domain. (**b**) Structural comparison of chains A and B in phyB^{Y276H}-PIF6β complex (PDB code: 9ITF in this study) and chains B and C in phyB^{Y276H}-908–PIF6 complex (PDB code: 9IUZ in Wang et al.[43]).

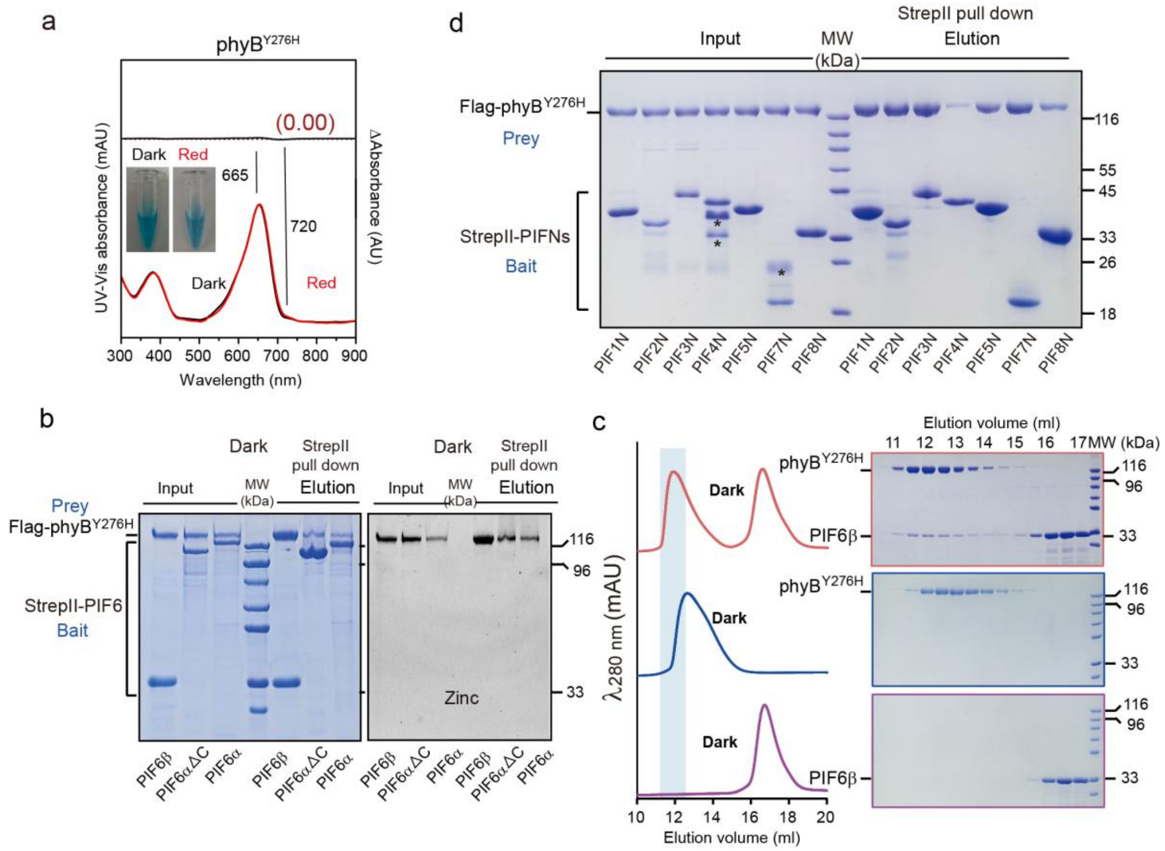

Fig. S7. phyB^{Y276H} is constitutively active and interact with PIFs in vitro. (**a**) UV-vis absorbance spectra of phyB^{Y276H} in the dark (dark line, Pr) and under red light irradiation (red line, Pfr). The Pr→Pfr difference spectrum is shown at 70% magnitude. The spectral change ratio is 0.00. Spectra are the average of three technical replicates. (**b**) The interactions between PIF6 and phyB^{Y276H} are assessed by pull-down assays. PIF6α, PIF6β, and PIF6αΔC are fused with a StrepII tag. PhyB^{Y276H} is fused with 3×Flag tag. Protein mixtures of indicated groups are incubated with Strep-Tactin Sepharose in the dark for 2 h. Samples in the "Input" and "Elution" were subjected to SDS-PAGE and gels were stained for protein with Coomassie blue (left panel) or for covalently bound PΦB by zinc-induced fluorescence (right panel). (**c**) PIF6β interacts with phyB^{Y276H} in size exclusion chromatography (SEC) analyses. All SEC analyses are performed using a Superose^{TM} 6 increase 10/300 GL column. Left panel shows the peak fractions of proteins in SEC. The absorbance at 280 nm is detected. The shaded area denotes the peak fractions of phyB^{Y276H}–PIF6β complex that are co-migrated. Right panel shows the SDS-PAGEs of peak fractions in left panel at the same elution volume from individual injections stained by Coomassie blue. (**d**) The interactions between phyB^{Y276H} and PIFNs are assessed by pull-down assays in the dark. All PIFNs are fused with a StrepII tag, while phyB^{Y276H} is fused with 3×Flag tag. Protein mixtures containing phyB^{Y276H} and different PIFNs in the "Input" are incubated with Strep-Tactin Sepharose in the dark for 2 h.

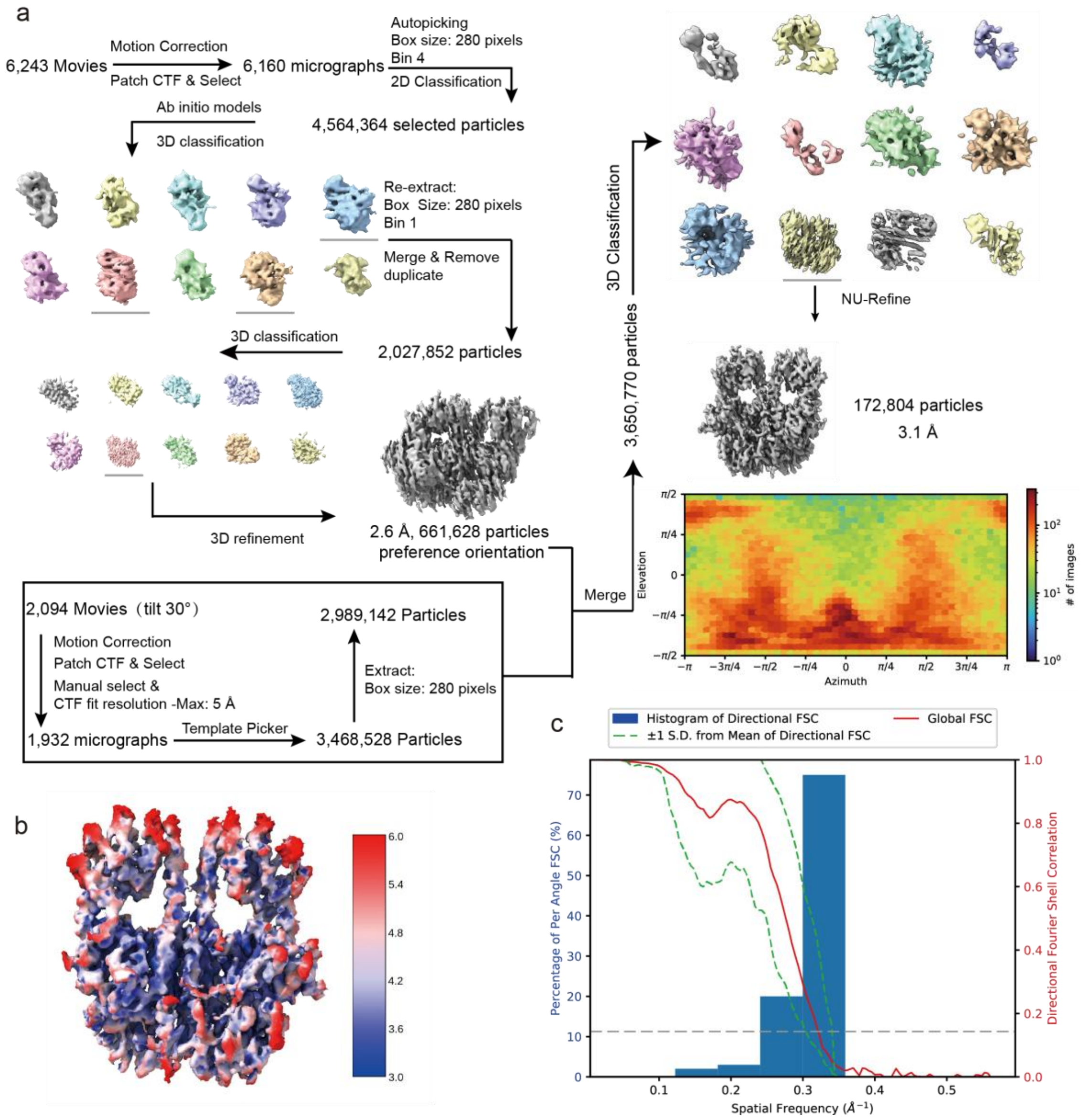

Fig. S8. Cryo-EM data processing of the phyB^{Y276H}–PIF6β complex. (**a**) Cryo-EM data processing workflow. (**b**) Local resolution for the consensus map. (**c**) 3D FSC curve of the phyB^{Y276H}–PIF6β complex EM map.

5

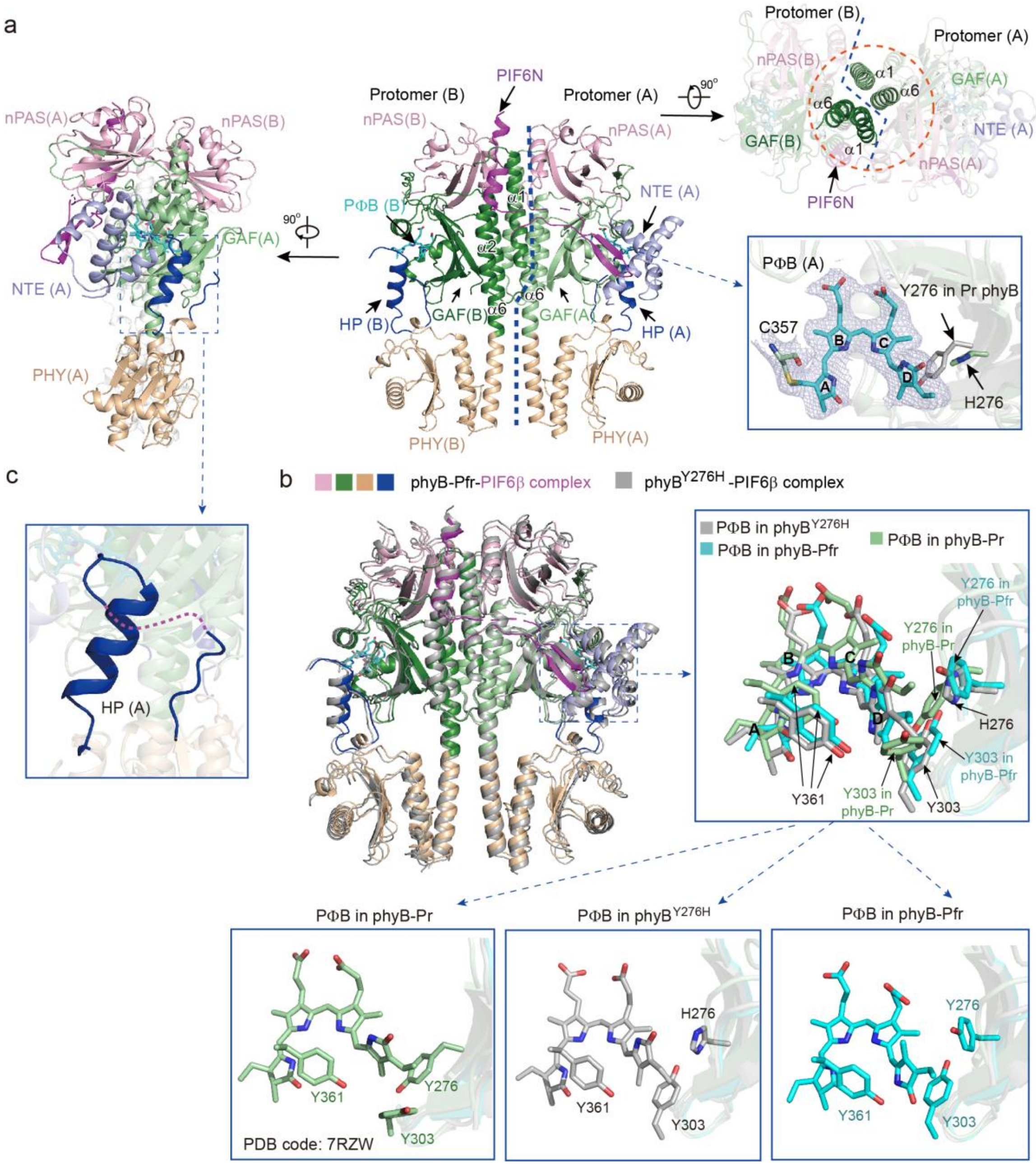

Fig. S9. Cryo-EM structures of the phyB^{Y276H}–PIF6β complex. (**a**) The overall structure of the full-length phyB^{Y276H}–PIF6β complex. Two phyB^{Y276H} molecules interact with one PIF6β molecule. Only PIF6N and phyB^{Y276H} PSMs are clearly modelled. Two phyB^{Y276H} PSMs assemble in a "head-to-head" manner. Structural overviews of the full-length phyB^{Y276H}–PIF6β complex in front (middle), top (upper right), and side (left) views are shown. The lower right shows the PΦB chromophore in phyB^{Y276H} protomer A. PΦB is covalently bound by C357. Domains in protomer A are labelled with "(A)"; domains in protomer B are labelled with "(B)". The orange dashed circle represents the four-helix bundle composed of helices α1 and α6 from two protomers. (**b**) Structural comparison of phyB-Pfr–PIF6β complex and phyB^{Y276H}–PIF6β complex. Right panel shows the superposition of PΦB chromophores in phyB-Pfr–PIF6β complex, phyB^{Y276H}–PIF6β complex, and phyB-Pr (PDB code: 7RZW)[39]. The D-ring of PΦB is in an "*E*" configuration

in phyB-Pfr and a “*Z*” configuration in phyB-Pr and phyB^{Y276H}. The D-rings of these PΦB chromophores are surround by several bulky aromatic residues (Y276, Y303, and Y361. (**c**) The hairpin loops (HPs) in phyB^{Y276H}–PIF6β complex fold into α helices.

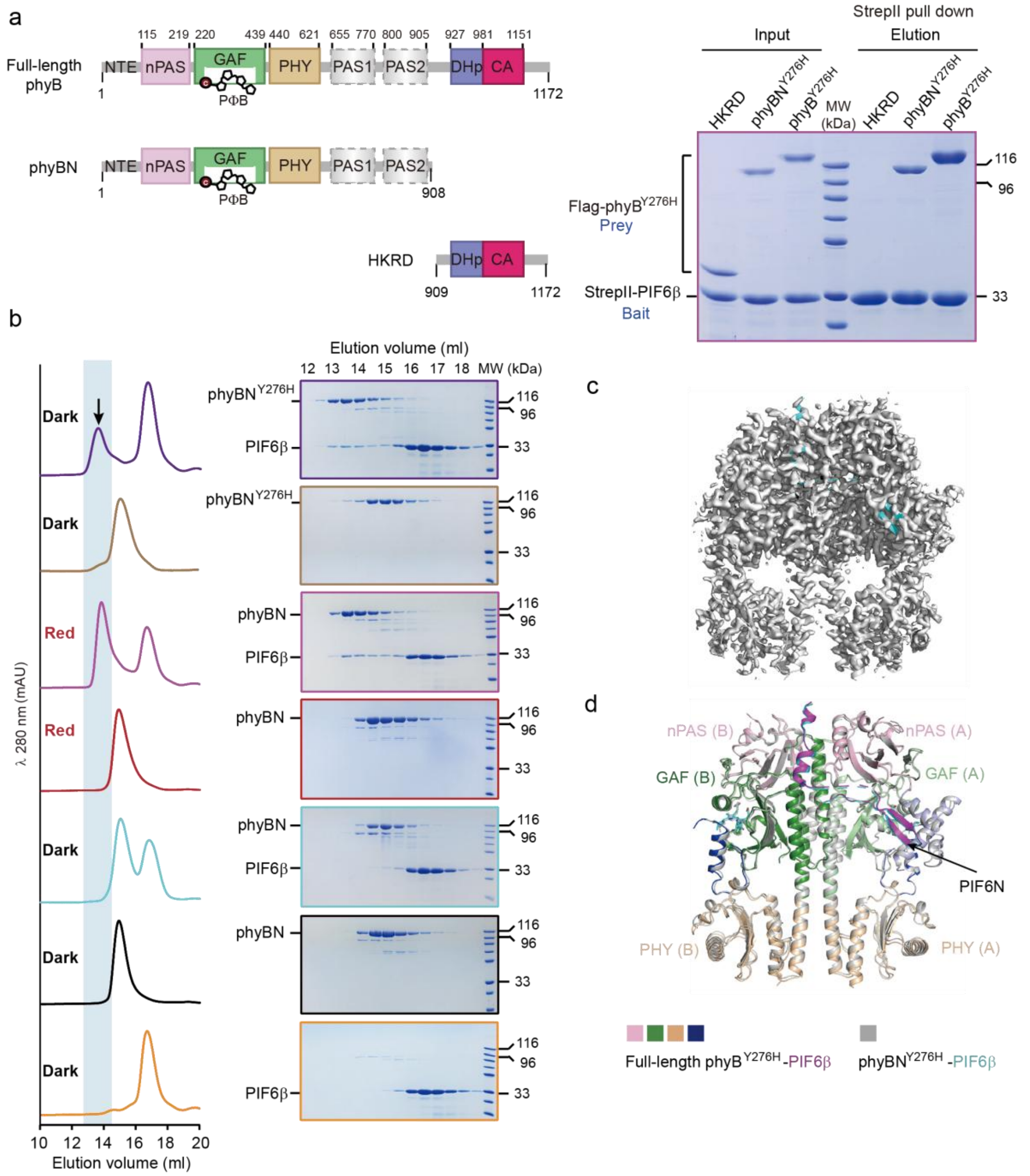

Fig. S10. phyBN is essential for PIF6β binding. (**a**) Left panel: Domain organization of full-length phyB and its truncations that lack the HKRD region (phyBN, 1–908 fragment) or the N-terminal region (HKRD, 909–1172 fragment). The numbers delineate domain boundaries within the phyB fragments. HKRD, histidine kinase-related domain. Right panel: Interactions between PIF6β and full-length phyB^{Y276H}, phyBN^{Y276H}, and HKRD are assessed by pull-down assays conducted in the dark. PIF6β is fused with a StrepII tag; The phyB^{Y276H} and phyBN^{Y276H} are fused with 3×Flag tag; The HKRD is fused with 6×His tag. Protein mixtures that contain PIF6β and different phyB^{Y276H} fragments in the "Input" are incubated with Strep-Tactin Sepharose in darkness for 2 h. kDa, kilodaltons. MW, molecular weight marker. (**b**) PIF6β interacts with phyBN^{Y276H} and red light-irradiated phyBN in size exclusion chromatography (SEC) analyses. All SEC analyses are performed using a Superose™ 6 increase 10/300 GL column. Left panel shows the peak fractions

of proteins in SEC. The absorbance at 280 nm is detected. The arrow and the shaded area denote the peak fractions of phyBN^{Y276H} (or red light-irradiated phyBN) –PIF6β complex that are co-migrated. Right panel shows the SDS-PAGEs of the peak fractions from the left panel at the same elution volume from individual injections stained by Coomassie blue. (**c**) Surface representation of the cryo-EM density map of phyBN^{Y276H}–PIF6β complex. Only the photosensory modules (PSMs) in phyBN^{Y276H} (grey) and N-terminus of PIF6β (PIF6N, cyan) are well traced in the phyBN^{Y276H}–PIF6β complex. (**d**) Structural comparison of full-length phyB^{Y276H}–PIF6β complex and phyBN^{Y276H}–PIF6β complex. In the full-length phyB^{Y276H}–PIF6β complex, nPAS, GAF, PHY, and PIF6N are colored in light pink, green, wheat, and magenta, respectively. In the phyBN^{Y276H}–PIF6β complex, PIF6N is shown in cyan, and the domains in phyBN^{Y276H} are shown in grey.

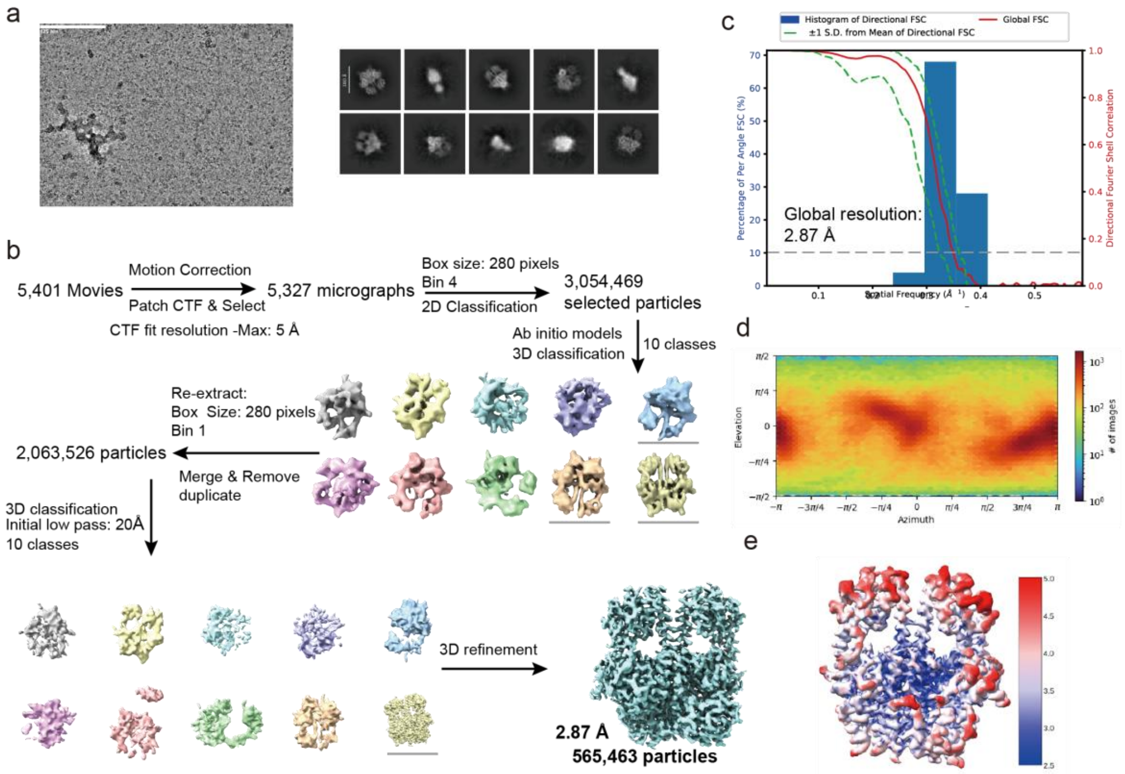

Fig. S11. Cryo-EM data processing of the phyBN^{Y276H}–PIF6β complex. (**a**) Representative micrograph and 2D class averages of the phyBN^{Y276H}–PIF6β complex. (**b**) Flowchart for the cryo-EM data processing of the phyBN^{Y276H}–PIF6β complex. (**c**) 3D FSC curves of 3D reconstruction. (**d**) Orientation distribution plot for the final reconstruction. (**e**) Local resolution for the consensus map. PhyBN, 1–908 fragment of phyB.

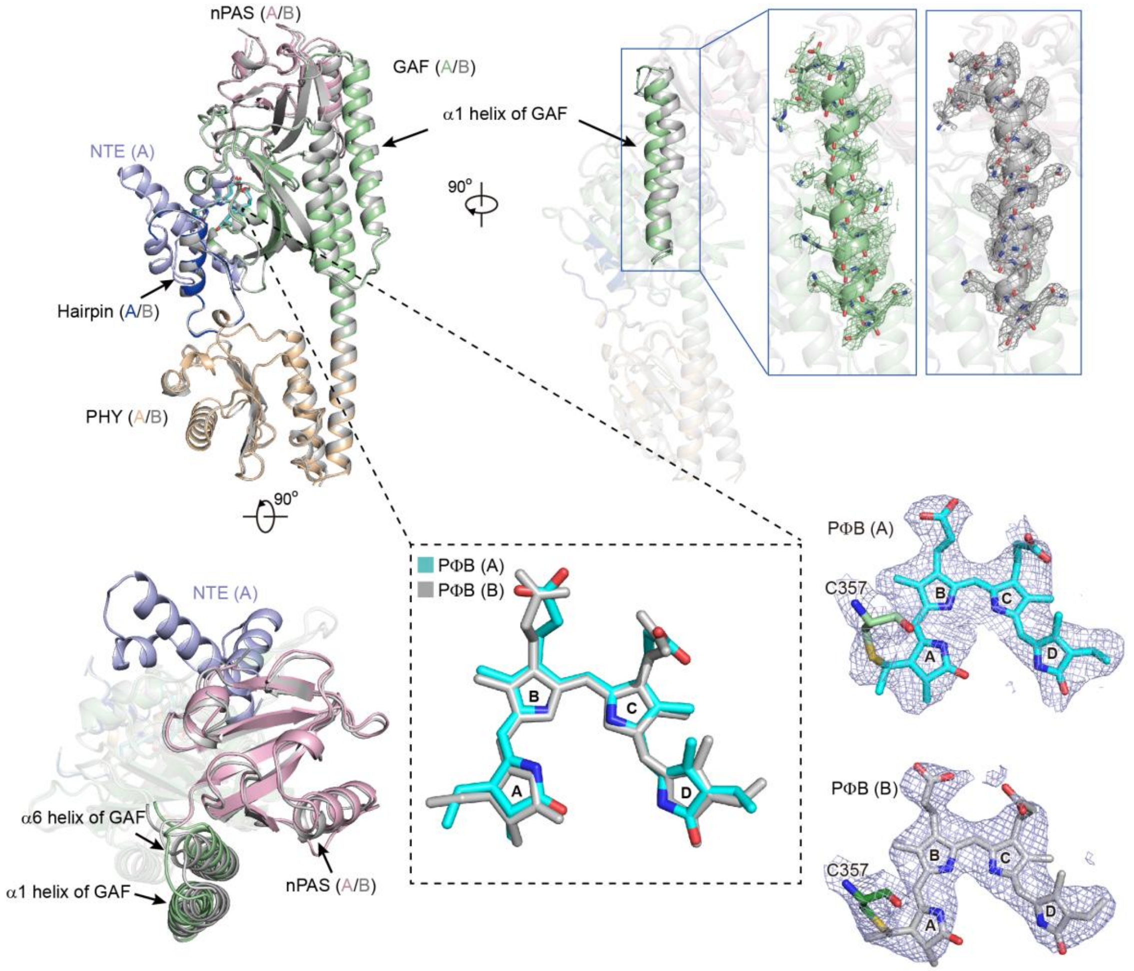

Fig. S12. Superposition of phyB-Pfr protomer A and protomer B in phyB-Pfr–PIF6β complex. The NTE, nPAS, GAF, and PHY domains in phyB-Pfr protomer A are colored light blue, light pink, light green, and wheat, respectively. All domains in phyB-Pfr protomer B are shown in grey. PΦB contains four pyrrole rings (A–D). PΦB in protomer A is shown in cyan, and PΦB in protomer B is shown in grey. The structures of the two phyB-Pfr protomers in phyB-Pfr–PIF6β complex are highly similar, with only slightly conformational changes observed in the nPAS domain and helices α1 and α6 of GAF domain. Cryo-EM densities of helices α1 and PΦB in protomers A and B are shown.

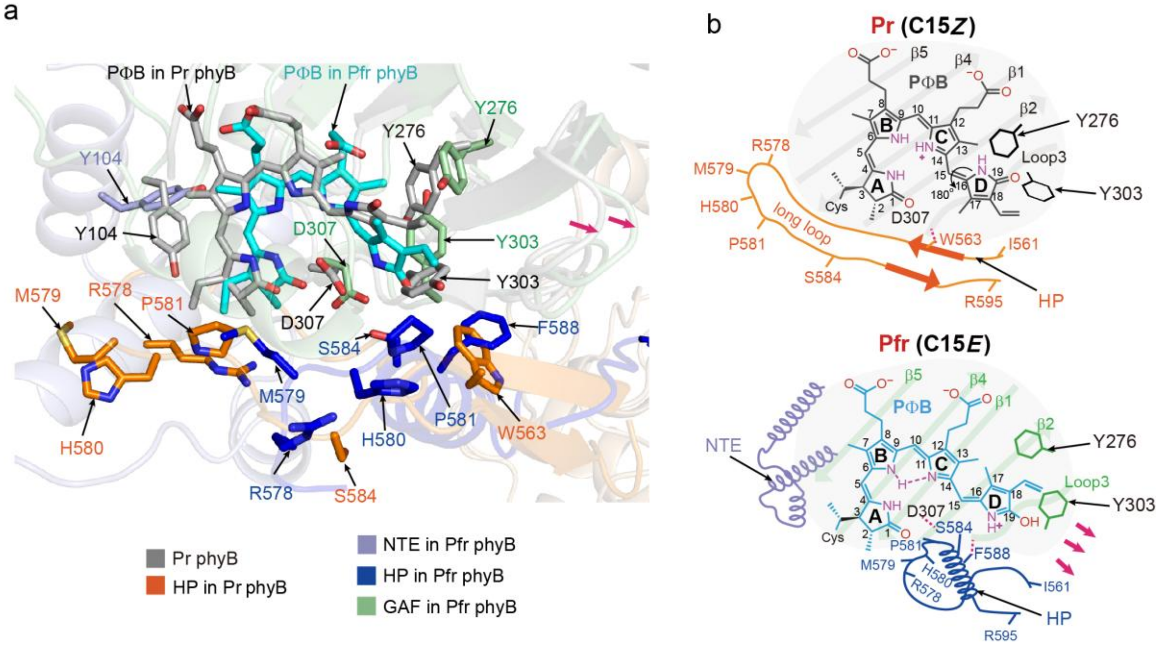

Fig. S13. Structural comparison of the PΦB binding pockets of phyB-Pr (PDB code: 7RZW) and PIF6β-bound phyB-Pfr. (**a**) Superposition of the PΦB binding pockets of protomer A in phyB-Pr (PDB code: 7RZW) and protomer A of PIF6β-bound phyB-Pfr. PΦB and its surrounding residues in phyB-Pr are shown in grey. Residues in the hairpin loop (HP) of phyB-Pr are shown in orange. PΦB in phyB-Pfr is shown in cyan. Residues from the NTE, GAF, and HP in phyB-Pfr are colored light blue, light green, and blue, respectively. Magenta arrows indicate the movements of corresponding β-sheets in the PΦB binding pocket of PIF6β-bound phyB-Pfr. The side chains of residues Y104, Y303, Y276, and D307 exhibit changes in orientation. (**b**) Proposed models that show the conformational changes in the PΦB binding pocket during Pr→Pfr photoconversion of plant phyB. Red light triggers a "*Z*" to "*E*" isomerization of the C15–C16 double bond between the C and D rings in PΦB. The side chains of several residues, including Y104, Y303, Y276, and D307, change their orientations. The HP loop folds into an α-helix, and its original position is occupied by the folded NTE.

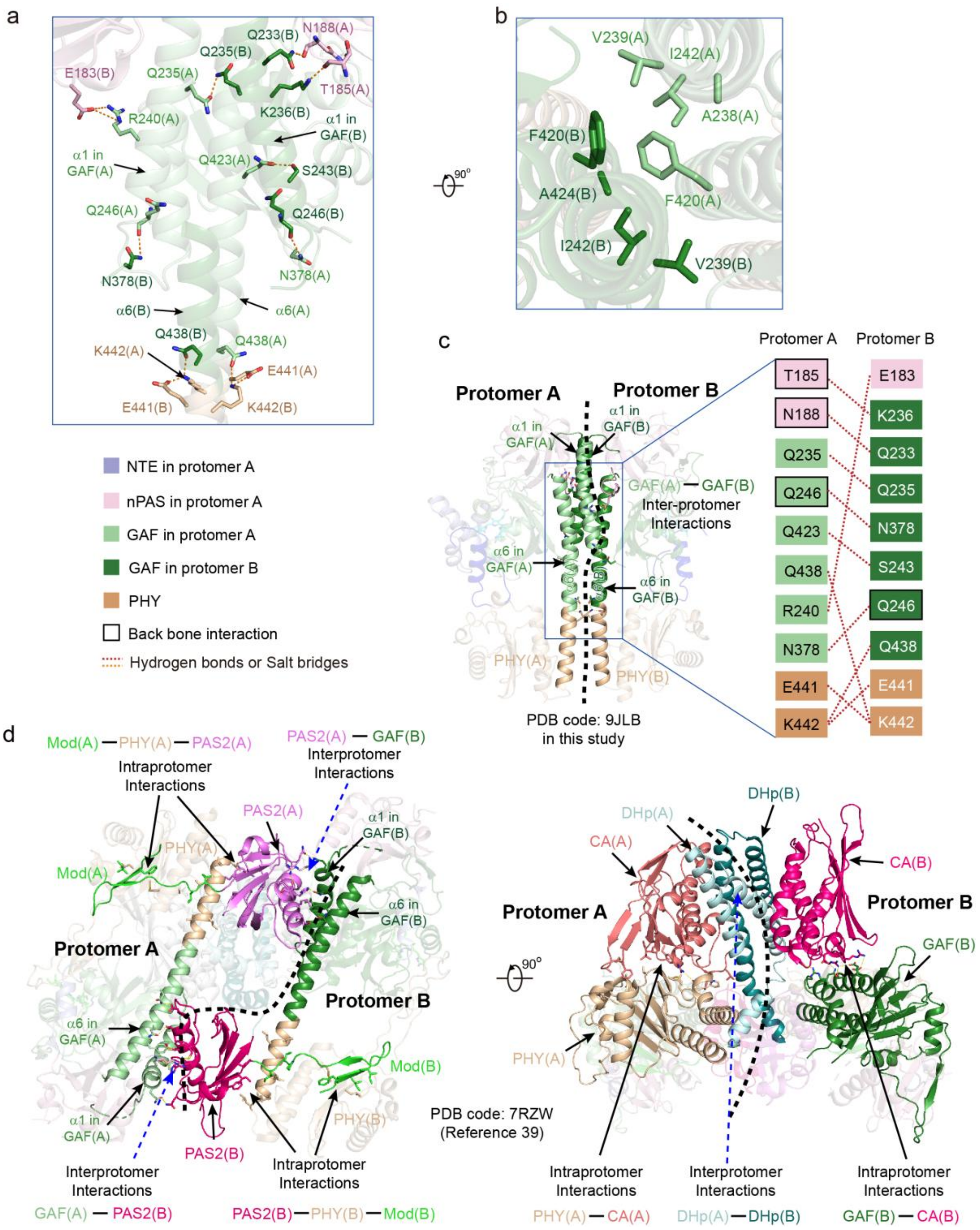

Fig. S14. Changes in the binding interfaces between phyB-Pr and phyB-Pfr. (**a**) Close-up view of the photosensory module (PSM) dimer interface in the PIF6β-bound phyB-Pfr. The interactions mainly occur between the α1 and α6 helices of the GAF domains in protomer A and protomer B. Residues involved in the dimerization are displayed as sticks and labelled with corresponding residue numbers. Residues in protomer A are labelled with "(A)"; Residues in protomer B are labelled with "(B)". The orange dashed lines indicate hydrogen bonds or salt bridges. (**b**) Hydrophobic interactions in the PIF6β-bound phyB-Pfr PSM dimer. (**c**)

Schematic representation of the interaction interfaces of PIF6β-bound phyB-Pfr PSM dimer. Left panel shows the interactions between phyB-Pfr protomer A and protomer B. The interactions mainly occur between the α1 and α6 helices of the GAF domains in protomer A and protomer B. (**d**) Intraprotomer and interprotomer interfaces in phyB-Pr dimer (PDB code: 7RZW[39]). Intraprotomer interactions are indicated by solid black arrows. Interprotomer interactions are indicated by dashed blue arrows. Mod, PAS2, CA, and DHp regions in phyB-Pfr (PDB code: 9JLB in this study) are invisible. GAF, cGMP phosphodiesterase/adenylyl cyclase/FhlA domain; PHY, phytochrome-specific domain; PAS2, Period/Arnt/Single-Minded domain; DHp, dimerization histidine phosphotransfer; CA, catalytic ATP-binding.

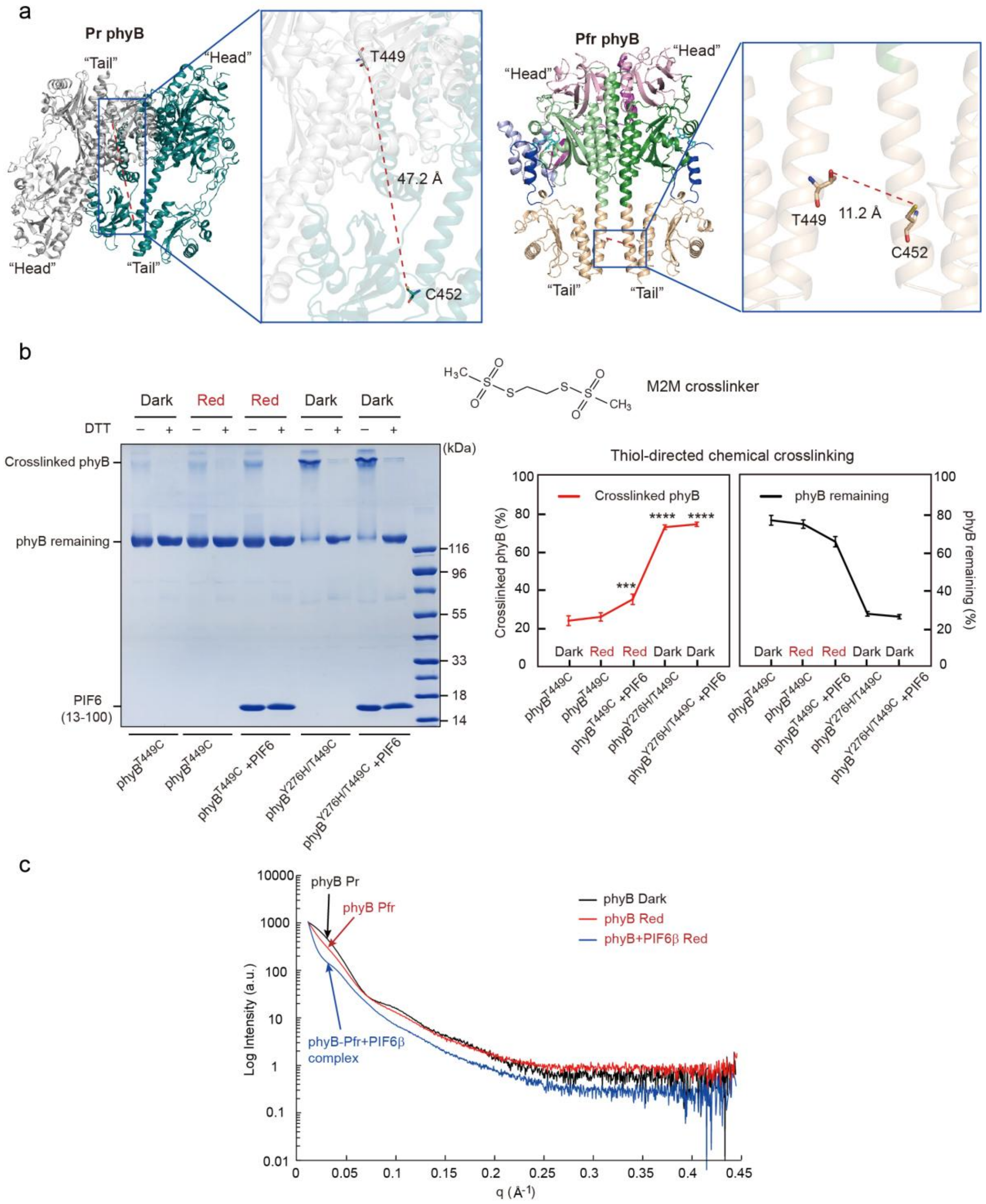

Fig. S15. Thiol-directed chemical crosslinking validates PSMs reassembly during photoactivation. (**a**) The distance between T449 and C452 in phyB-Pr is 47.2 Å, while in phyB-Pfr is 11.2 Å. (**b**) SDS-PAGE imaging of thiol-directed chemical crosslinking. The 1,2-ethanediyl bismethanethiosulfonate (M2M) crosslinker can crosslink two cysteine residues within a Cβ-Cβ distance of ~11 Å. We mutated T449 in phyB or phyB^{Y276H} to C449. To avoid the nonspecific crosslink, we also introduced C925S, C936S, C972S, and C1121S into phyB^{T449C} and phyB$^{Y276H/T449C}$. In the dark, C449 (mutated from T449) and C452 are far apart (47.2 Å) and therefore are difficult to be crosslinked. After red light irradiation or in phyB^{Y276H}, C449 and

C452 are easily crosslinked by M2M, and the crosslinked phyB can be reversibly reduced by DTT (dithiothreitol). The addition of PIF6 N-terminal fragment (residues 13–100) led to a significant increase in the amount of cross-linked bands. The right panel shows the quantification of the results in the left panel. The integrated densities of the bands of the "crosslinked phyB" (A) and "phyB remaining" (B) are measured using Image J software. Cross-linked phyB (%) is calculated as A/A+B. PhyB remaining (%) is calculated as B/A+B. The standard deviation is derived from four measurements. Data are presented as mean±SD. ***, $P<0.001$; ****, $P<0.0001$. (**c**) Conformational changes of phyB-Pr (dark line), phyB-Pfr (red line), and phyB-Pfr–PIF6β complex (blue line) examined by SAXS.

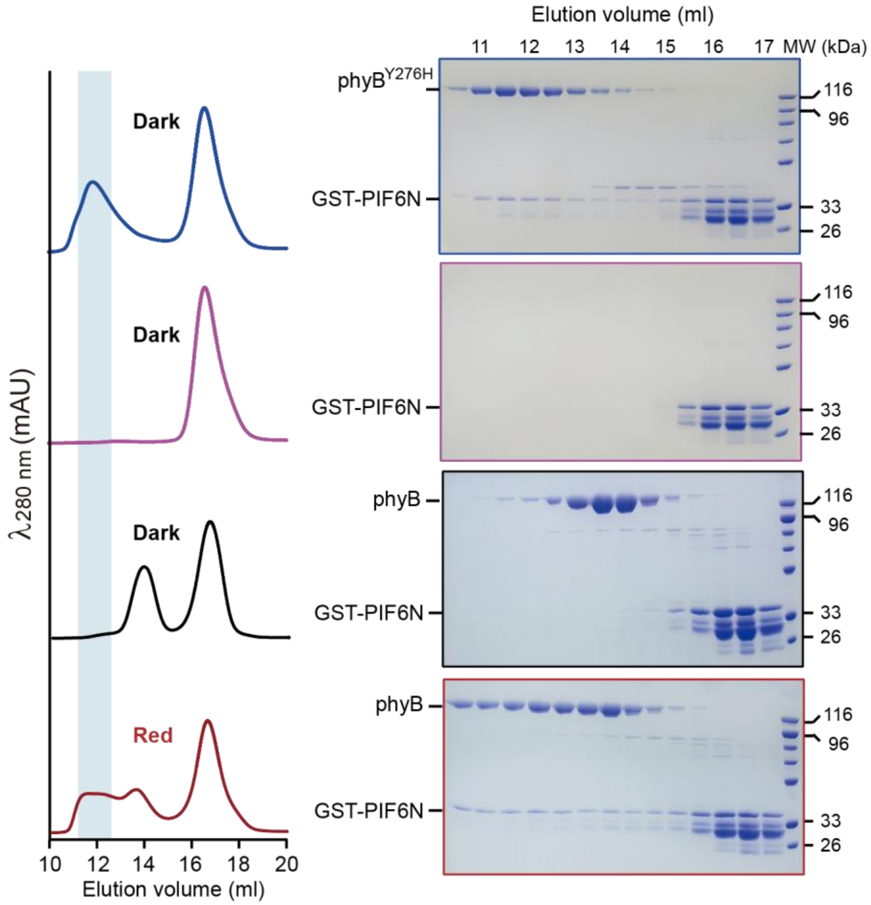

Fig. S16. PIF6N is sufficient for phyB binding. PIF6N (N-terminus of PIF6β, residues 1–64) is fused to a glutathione S-transferase (GST) tag. The interactions between GST-PIF6N and full-length phyB or phyB^{Y276H} are assessed by size exclusion chromatography (SEC) under red light irradiation or in the dark. The absorbance at 280 nm is detected. The shaded area denotes the peak fractions of the phyB-Pfr- or phyB^{Y276H}–PIF6N complex that are co-migrated. kDa, kilodaltons; MW, molecular weight marker.

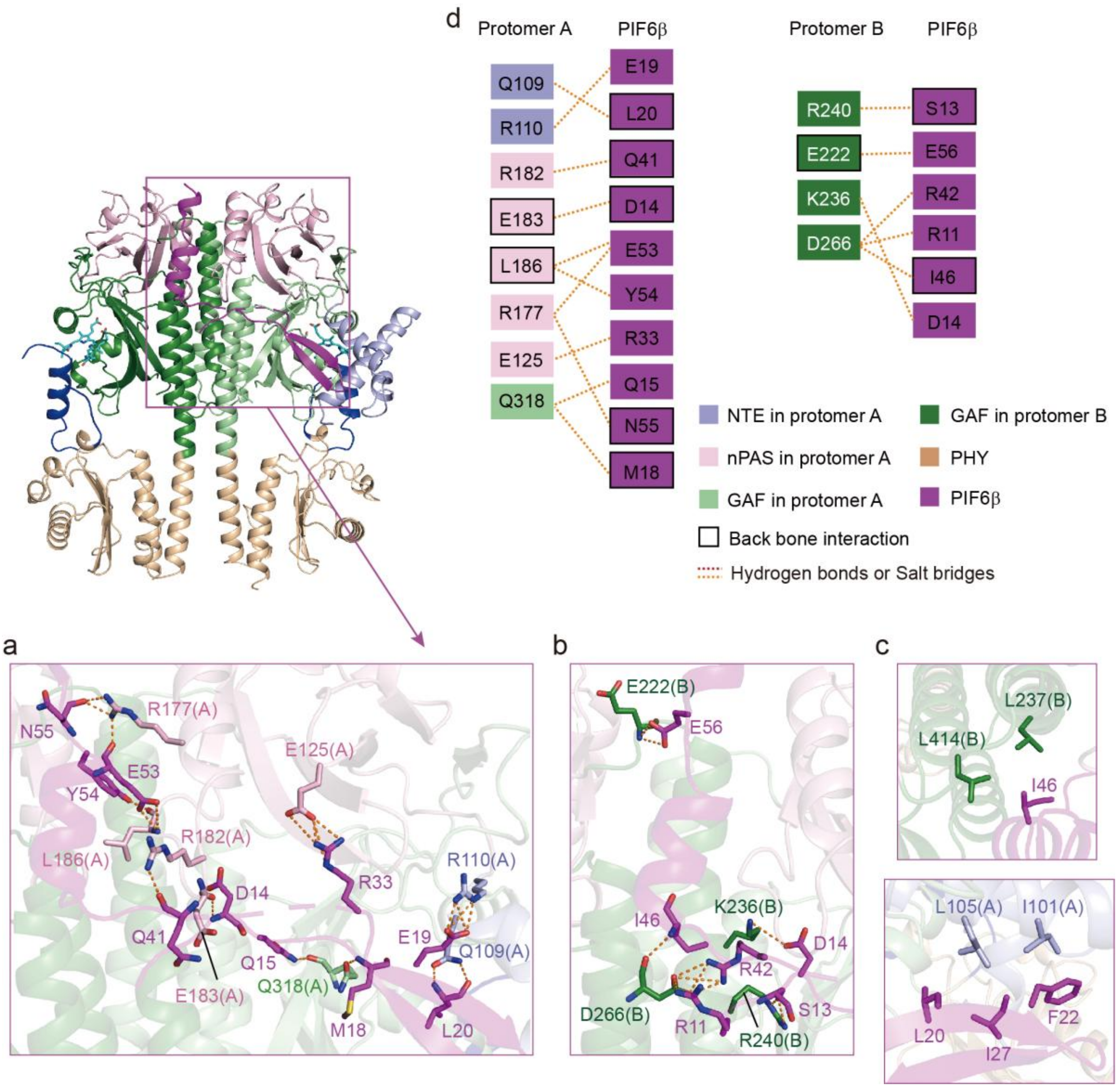

Fig. S17. Binding interfaces of the phyB-Pfr–PIF6β complex. (**a**) Close-up view of the binding interface between phyB-Pfr protomer A and PIF6N. The orange dashed lines indicate hydrogen bonds or salt bridges. PIF6N: N-terminus of PIF6β. (**b**) Close-up view of the binding interface between phyB-Pfr protomer B and PIF6N. (**c**) Hydrophobic interactions between phyB-Pfr and PIF6N. (**d**) Schematic representation of the interaction interfaces of phyB-Pfr–PIF6β complex. Left panel shows the interactions between phyB-Pfr protomer A and PIF6β. Right panel shows the interactions between phyB-Pfr protomer B and PIF6β. The black square outlines indicate that the main chain of these amino acids is involved in the interactions. The orange dashed lines indicate hydrogen bonds or salt bridges.

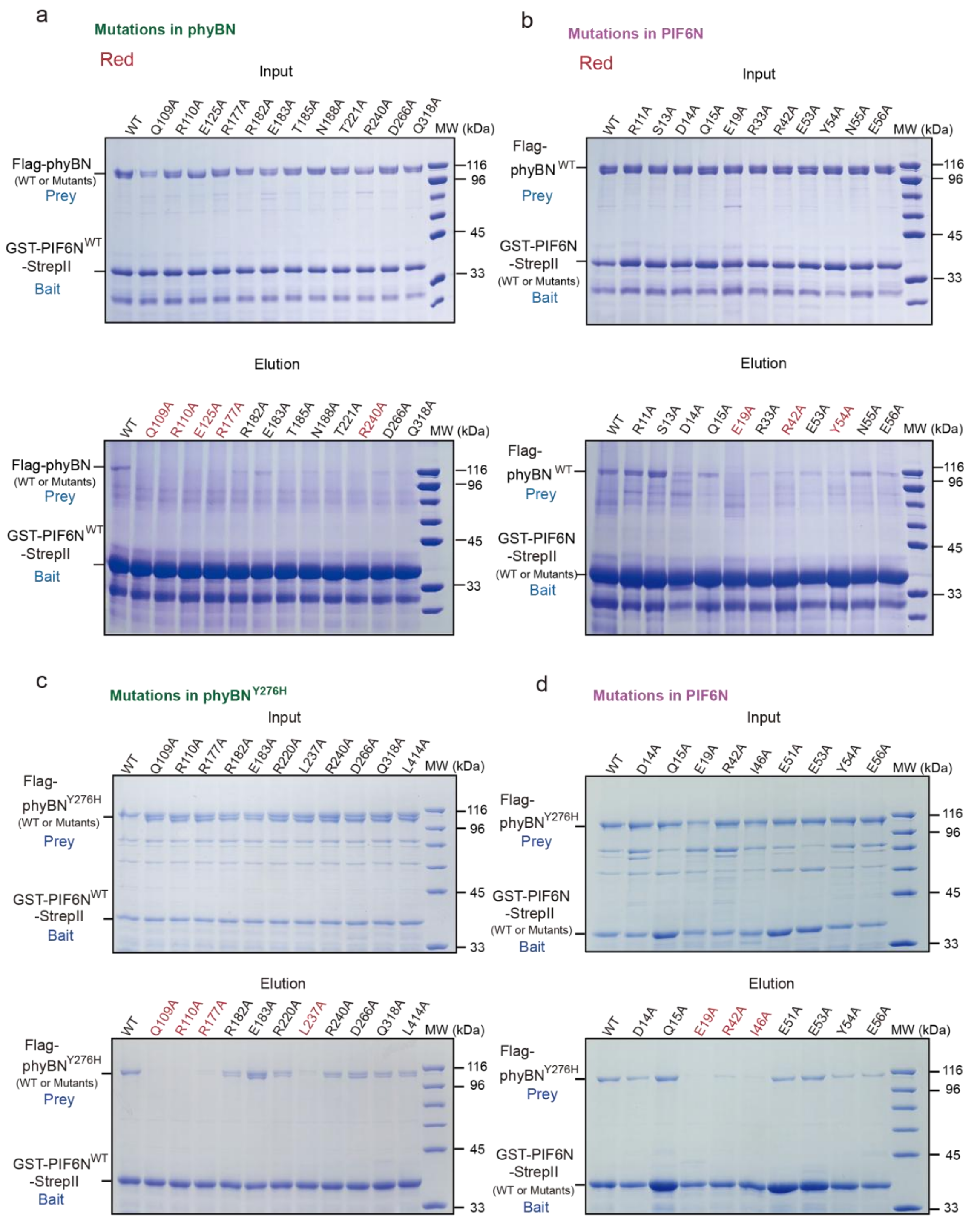

Fig. S18. Mutagenesis analysis of phyB-Pfr (or phyB^{Y276H}) and PIF6β binding interfaces. The PDB file of the full-length phyB-Pfr–PIF6β complex or phyB^{Y276H}–PIF6β complex is loaded to PDBePISA (https://www.ebi.ac.uk/pdbe/pisa/) to analyze the interface between phyB and PIF6β. Residues with a higher buried area percentage in the PDBePISA interface analysis might make greater contributions to the interaction. Residues with the highest accessible surface area (ASA) and buried surface area (BSA) in phyB and in PIF6β will be mutated to assess their contributions in mediating the interactions between phyB and PIF6β. (**a and c**) Analysis of the interactions between wild-type PIF6N (PIF6N^{WT}) and different phyBN or

phyBN^{Y276H} mutants. Residues in phyBN or phyBN^{Y276H} that interact with PIF6N via their side chains were mutated. Animo acids Q109, R110, R177, R182, E183, R220, L237, R240, D266, Q318, and L414 in phyBN or phyBN^{Y276H} are mutated to A, respectively. The interactions between PIF6N^{WT} and phyBN or phyBN^{Y276H} mutants are assessed by pull-down assays under red light irradiation or in the dark. PIF6N^{WT} is fused with a glutathione S-transferase (GST) tag at N-terminus and a StrepII tag at C-terminus. PhyBN or phyBN^{Y276H} mutants are fused with 3×Flag tag. Protein mixtures that contain PIF6N^{WT} and PhyBN (or phyBN^{Y276H}) mutants in the "Input" are incubated with Strep-Tactin Sepharose under red light or in the dark. (**b and d**) Interactions analysis of different PIF6N mutants with phyBN or phyBN^{Y276H}. Residues of PIF6N that interact with phyBN or phyBN^{Y276H} via their side chains were mutated. Animo acids D14, Q15, E19, R42, I46, E51, E53, Y54, and E56 in PIF6N are mutated to A, respectively. The interactions between PIF6N mutants and phyBN or phyBN^{Y276H} are assessed by pull-down assays under red light irradiation or in the dark. All PIF6N mutants are fused with a GST tag at N-terminus and a StrepII tag at C-terminus. PhyBN or phyBN^{Y276H} is fused with 3×Flag tag. Protein mixtures containing different PIF6N mutants and phyBN or phyBN^{Y276H} in the "Input" are respectively incubated with Strep-Tactin Sepharose under red light or in the dark. Residues critical for complex formation are shown in red. PIF6N, N-terminus of PIF6β (residues 1–64); phyBN, 1–908 fragment of phyB.

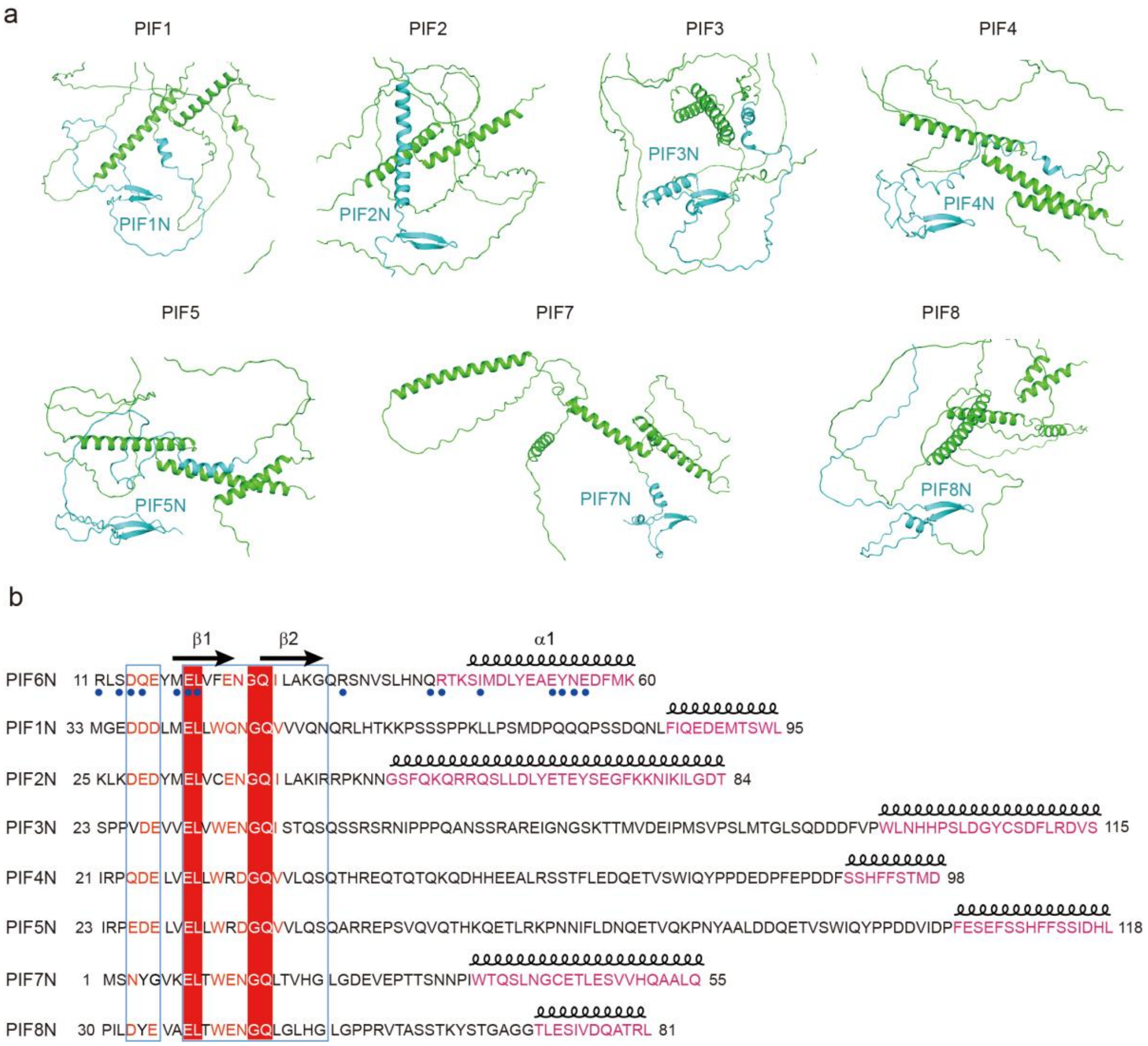

Fig. S19. N termini of other PIFs in *Arabidopsis* might adopt a similar fold to PIF6N. (**a**) AlphaFold 2-predicted structures of PIF1–PIF5, PIF7–PIF8 in *Arabidopsis*. N termini that contain a pair of β-sheets and an α-helix in different PIFs are shown in cyan. (**b**) On the basis of the structure of PIF6N that we have determined and the structures of the other *Arabidopsis* PIFs predicted by AlphaFold 2 in (**a**), we have speculated the regions in other PIFs that might interact with phyB-Pfr. Secondary structural elements are shown above the sequence. The numbers of amino acids are marked at both ends of the sequence. Sequence identity is indicated by white letters on a red background, and sequence similarity is indicated by red letters. The dark blue dots below the sequence of PIF6N indicate residues involved in phyB^{Y276H} interactions. APB motif, active phyB-binding motif.

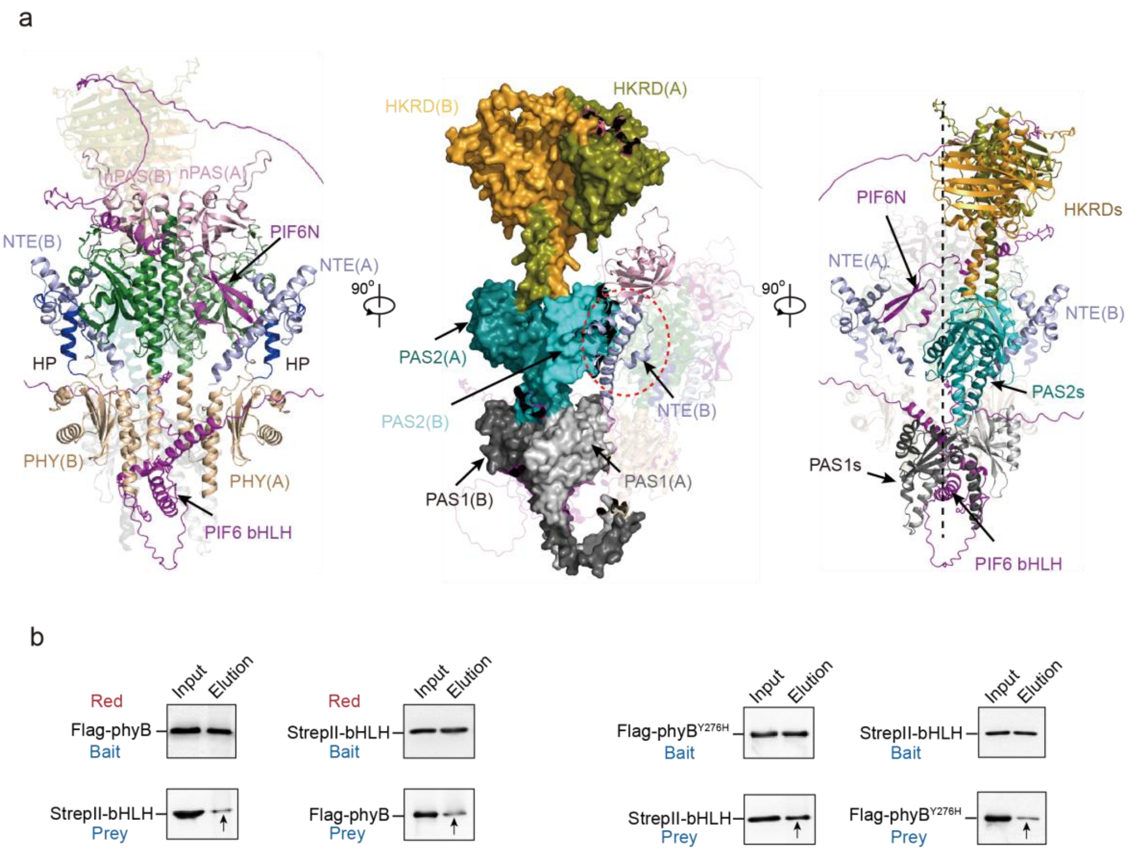

Fig. S20. The 3D structure of the full-length phyB^{Y276H}–PIF6α complex predicted by AlphaFold 3. (**a**) The 3D structure of full-length phyB^{Y276H}–PIF6α complex predicted by AlphaFold3. Domains in protomer A are labelled with "(A)"; Domains in protomer B are labelled with "(B)". Left panel shows the photosensory modules (PSMs) of phyB^{Y276H} which contain the NTE (light blue), nPAS domain (light pink), GAF domain (light or dark green), and PHY domain (wheat). Two PSMs assemble in a "head-to-head" manner. PIF6α is shown in magenta. The N-terminus of PIF6α (PIF6N) contains a pair of β-sheets and an α-helix. The basic helix-loop-helix (bHLH) domain of PIF6α interacts with the PHY domain of phyB^{Y276H}. Middle and right panels show the output module of phyB^{Y276H}, containing the PAS1, PAS2, and HKRD domains. Two output modules form a "head-to-head" dimer. The PAS2 domain of phyB^{Y276H} protomer B is close to its NTE (indicated by the red dashed circle), which might inhibit the binding of the second PIF6 molecule to phyB^{Y276H}. (**b**) The interactions between bHLH domain of PIF6α and phyB or phyB^{Y276H} are assessed by pull-down assays under red light irradiation or in the dark. The bHLH domain of PIF6α is fused with a StrepII tag. PhyB or phyB^{Y276H} are fused with 3×Flag tag. Protein mixtures containing phyB or phyB^{Y276H} and bHLH in the "Input" are incubated with StrepII or Flag beads under red light irradiation or in the dark. Bovine serum albumin (BSA) is included in the "Input" to suppress non-specific interactions. All "Input" samples are diluted by a factor of 10 before being applied to the western blot assays.

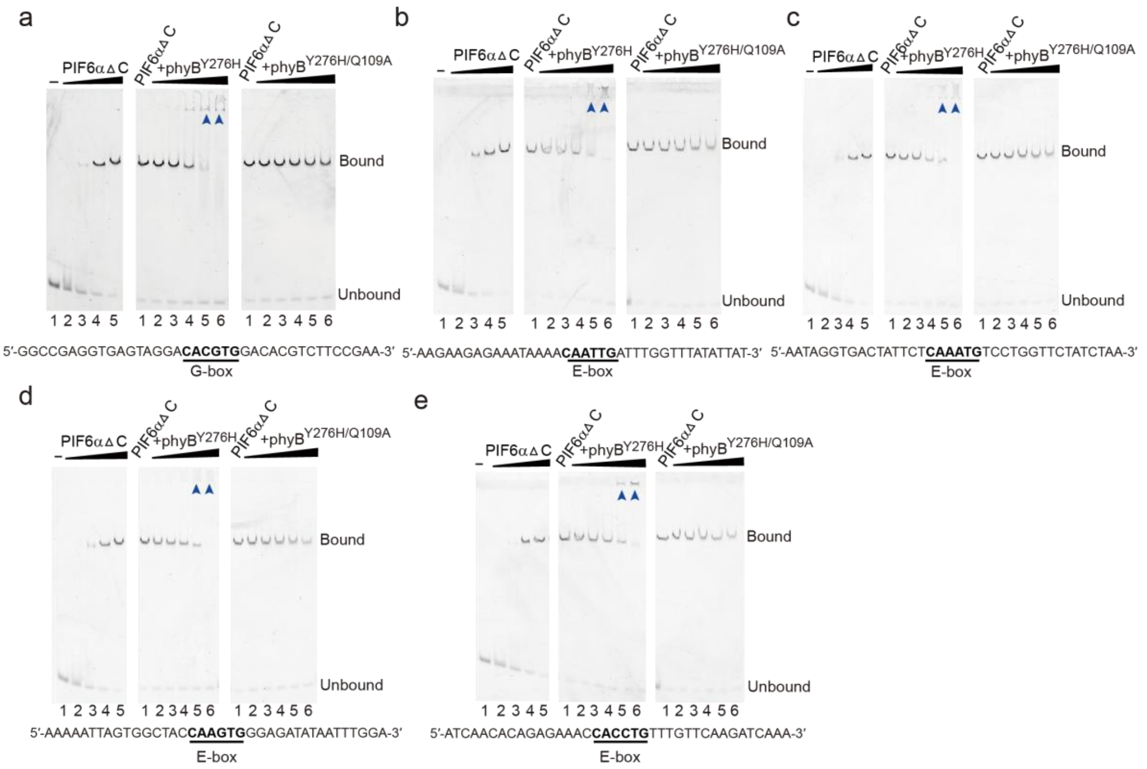

Fig. S21. PhyB^{Y276H} influences the DNA-binding activity of PIF6. Electrophoretic mobility shift assay (EMSA) analysis shows that PIF6αΔC can bind to the DNA probe containing the G-box (**a**) and E-box (**b–e**) motifs. (**a**) PIF6αΔC can bind to a reported G-box containing DNA probe "G-wt"[30]. (**b–e**) PIF6αΔC can bind to different E-box containing elements of *FT* protomer[25]. The DNA probe is labeled with FAM at the 5′ end. Left panel: the final DNA probe concentrations in lane 1 to 5 are all 15 nM, and the corresponding protein concentrations are 0, 0.375, 0.75, 1.5, and 3.0 μM, respectively. Middle and right panels: phyB^{Y276H} influences the DNA binding activity of PIF6αΔC. The final concentrations of PIF6αΔC in lane 1 to 6 are all 3.0 μM, and the corresponding concentrations of phyB^{Y276H} or phyB$^{Y276H/Q109A}$ are 0, 0.75, 1.5, 3.0, 4.5, and 6.0 μM, respectively. PhyB$^{Y276H/Q109A}$: the mutant that almost loses interaction with PIF6N.

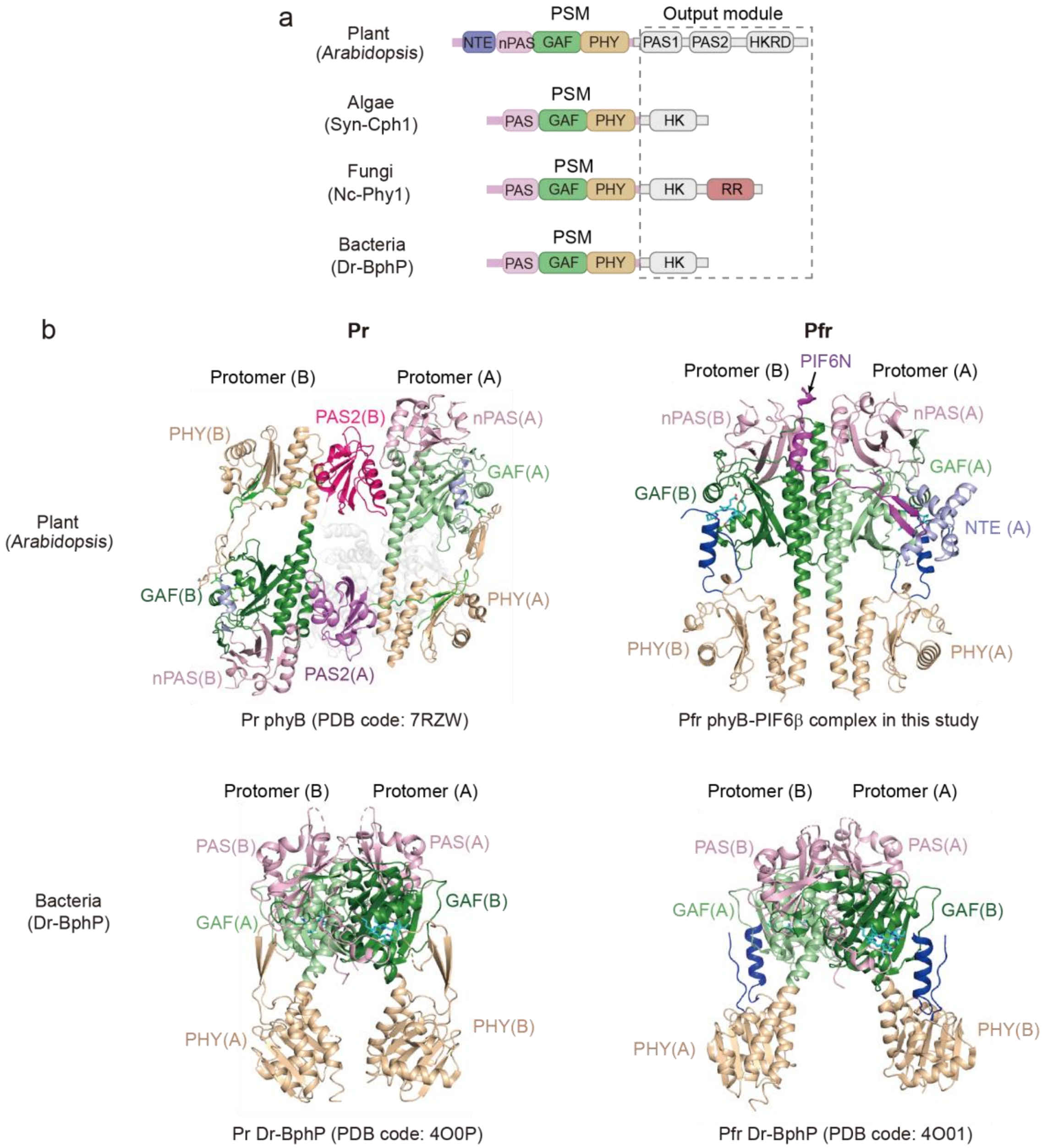

Fig. S22. The Pr→Pfr conversion of plant phytochrome B and bacteria phytochrome. (**a**) Domain architecture of representative phytochromes in plants, algae, fungi, and bacteria[55]. Canonical phys retain the conserved photosensory modules (PSMs), but possess relatively diverse output modules. *Syn*-Cph1, *Synechocystis* PCC6803 (Syn) Cph1; *Nc*-Phy1, *Neurospora crassa* (Nc) Phy1; *Dr*-BphP, *D. radiodurans* (Dr) BphP; NTE, N-terminal extension; nPAS, N-terminal Period/Arnt/Sim domain; GAF, cGMP phosphodiesterase/adenylyl cyclase/FhlA domain; PHY, phytochrome-specific domain; HKRD, histidine kinase related domain; HK, histidine kinase; RR, response regulator. (**b**) Structures of *Arabidopsis* phyB and *Dr*-BphP in the Pr and Pfr forms[39,56]. Domains in protomer A are labelled with "(A)"; domains in protomer B are labelled with "(B)".

Table S1

	Wild type phyB–PIF6β complex (PDB ID, 9JLB) (EMDB ID, EMD-61582)	phyB^{Y276H}–PIF6β complex. (PDB ID, 9ITF) (EMDB ID, EMD-60860)	phyBN^{Y276H} (1–908)-PIF6β complex. (PDB ID, 9IRK) (EMDB ID, EMD-60816)
**Data collection and Processing**			
Microscope	Krios	Krios	Krios
Voltage(keV)	300	300	300
Camera	Gatan K3	Gatan K3	Gatan K3
Magnification	105,000	105,000	105,000
Pixel size at detector (Å/pixel)	0.824	0.84	0.85
Total electron exposure (e–/Å^2)	50	50	50
Exposure rate (e–/pixel/s)	15.938	15.938	15.000
Exposure time (s)	2.14	2.21	2.41
Number of frames collected during exposure	40	40	40
Defocus range (μm)	–1.0 ~–2.0	–1.0 ~ –2.0	–1.0 ~ –2.0
Automation software	EPU v2.9	EPU v2.9	EPU v2.9
Energy filter slit width	20 eV	20 eV	20 eV
Micrographs collected (no.)	5,769	6,243 (tilt 0°), 2,094 (tilt 30°)	5,401
Micrographs used (no.)	5,767	8,092	5,327
Total extracted particles (no.)	9,028,696	7,622,253	3,098,879
For each reconstruction:			
Refined particles (no.)	8,852,952	7,553,506	3,054,469
Final particles (no.)	429,631	172,804	565,463
Point-group	C1	C1	C1
Resolution (global, Å)			
FSC 0.5 (unmasked/masked)	3.7/3.2	4.2/3.5	3.9/3.1
FSC 0.143 (unmasked/masked)	3.4/3.0	3.8/3.1	3.2/2.8
Resolution range (local, Å)	2.5–5.0	3.0–6.0	2.5–5.0
Map sharpening *B* factor (Å^2)	165	107.9	146.9
Map sharpening methods	Half-maps correlation	Half-maps correlation	Half-maps correlation
**Model composition**			
Protein	1,052	1,007	1,052
Ligands/Nucleotide	2	2	2
**Model Refinement**			
Refinement package	PHENIX	PHENIX	PHENIX
- real or reciprocal space	Real space	Real space	Real space
- resolution cutoff	3.0	3.1	2.8
Model-Map scores			
- CCvolume/CCmask	0.81/0.84	0.78/0.89	0.81/0.85

B factors (Å^2)			
-Protein residues	56.07	87.28	49.50
-Ligand	37.92	70.17	25.83
R.m.s. deviations from ideal values			
-Bond lengths (Å)	0.004	0.005	0.005
-Bond angles (°)	0.584	0.676	0.687
**Validation**			
MolProbity score	1.26	1.82	1.35
CaBLAM outliers	1.68	2.16	1.78
Clashscore	4.60	6.82	2.79
Poor rotamers (%)	0.78	1.76	1.12
C-beta deviations	0	0	0
Average Q-score	0.555	0.524	0.583
EMRinger score	4.06	3.18	2.91
**Ramachandran plot**			
Favored (%)	97.87	96.17	96.32
Outliers (%)	0	0	0

Table S1. Cryo-EM data collection, refinement, and validation statistics for the full-length *Arabidopsis* phyB-Pfr–PIF6β complex, phyB^{Y276H}–PIF6β complex, and phyBN^{Y276H} (1–908) –PIF6β complex.

Table S2

88 µmol m^{-2} s^{-1}

Samples	Amp %	k_1 (min^{-1})	$\epsilon_{R665} \cdot \phi_R$
phyB	47.28±0.41	1.00±0.04	1.15×10^6
phyB+PIF6β	56.65±0.38	10.54±0.61	18.64×10^6
phyB+PIF6β$^{3\ mut}$	53.89±0.38	2.48±0.01	3.64×10^6

617 µmol m^{-2} s^{-1}

Samples	Amp %	k_1 (min^{-1})	k_2 (min^{-1})	$\epsilon_{R665} \cdot \phi_R$
phyB	44.18±0.64	1.59±0.22	0.33±0.07	0.19×10^6
phyB+PIF6β	55.91±0.14	27.92±2.24	4.50±0.41	6.40×10^6
phyB+PIF6β$^{3\ mut}$	52.08±0.61	2.95±0.24	0.37±0.08	0.46×10^6

867 µmol m^{-2} s^{-1}

Samples	Amp %	k_1 (min^{-1})	k_2 (min^{-1})	$\epsilon_{R665} \cdot \phi_R$
phyB	47.88±0.64	9.62±1.02	0.46±0.02	1.12×10^6
phyB+PIF6β	56.08±0.11	36.50±2.14	4.65±0.25	5.88×10^6
phyB+PIF6β$^{3\ mut}$	52.43±0.34	13.87±1.30	0.96±0.07	1.85×10^6

Table S2. Pr→Pfr photoconversion rate constants and quantum efficiency for phyB alone, phyB+PIF6β, and phyB+PIF6β$^{3\ mut}$ under different red light intensities. For the measurements of Pr→Pfr photoconversion, phyB alone, phyB+PIF6β, and phyB+PIF6β$^{3\ mut}$ samples were irradiated with far-red light (730 nm, 655 µmol m^{-2} s^{-1}) for ~10 minutes to ensure all phyB proteins remained in the Pr state. Then the samples were exposed to different red light intensities (665 nm, 88 µmol m^{-2} s^{-1}, 617 µmol m^{-2} s^{-1}, and 867 µmol m^{-2} s^{-1}) until they reached steady state. The absorbances at 665 nm and 720 nm were recorded at corresponding time intervals to evaluate the photoconversion of the samples. The kinetic rate constants for photoconversion were calculated using the equation: $Abs_i = \Delta Abs_i \cdot exp(-k \cdot t) + Abs(\infty)$, where i represents the i-th wavelength, Abs is the absorption, ΔAbs is the reaction amplitude (Amp), k is the rate constant, t is time, and Abs (∞) is the absorption at time zero[70]. If two exponentials were required to explain the results, the data were fit to a simple sum of two exponentials using the equation described above. The

photoconversion quantum efficiency (ϕ) was calculated according to Butler's equation[70–72]. The rate constants for these samples are presented as the mean±standard deviation.

Table S3

Pfr→Pr photoconversion rate constant for phyB, phyB+PIF6β, and phyB+PIF6β$^{3\ mut}$ under far-red light.

Samples	Amp %	k_1 (min^{-1})
phyB	77.82±8.44	9.59±1.52
phyB+PIF6β	71.79±4.32	8.57±2.30
phyB+PIF6β$^{3\ mut}$	76.50±1.31	9.29±0.94

Pfr→Pr thermal reversion rate constant at 25 °C for phyB, phyB+PIF6β, and phyB+PIF6β$^{3\ mut}$.

Samples	Amp %	k_1 (min^{-1})	k_2 (min^{-1})
phyB	93.45±0.66	1.75 (0.33) ×10^{-1}	2.21 (0.13) ×10^{-2}
phyB+PIF6β	14.33±8.31	4.80 (0.002) ×10^{-3}	4.80 (0.002) ×10^{-3}
phyB+PIF6β$^{3\ mut}$	54.21±5.20	1.14 (0.19) ×10^{-1}	3.64 (0.12) ×10^{-3}

Table S3. Pfr→Pr conversion rate constants for phyB alone, phyB+PIF6β, and phyB+PIF6β$^{3\ mut}$ under far-red light or in the dark. Detailed kinetic measurements of Pfr→Pr photoconversion of phyB, phyB+PIF6β, and phyB+PIF6β$^{3\ mut}$ under far-red light (730 nm, 655 μmol m^{-2} s^{-1}) or in the dark (thermal reversion at 25 °C). The absorbance at 665 nm and 720 nm was recorded at corresponding time intervals to evaluate the photoconversion of these samples. Pfr→Pr thermal reversion was measured at 25 °C in the dark after driving the Pfr levels to steady state with red light (665nm, 867 μmol m^{-2} s^{-1}). The kinetic rate constants for photoconversion and thermal reversion were calculated using the equation: $Abs_i = \Delta Abs_i \cdot \exp(-k \cdot t) + Abs(\infty)$, where i represents the i-th wavelength, Abs is the absorption, ΔAbs is the reaction amplitude, k is the rate constant, t is time, and Abs (∞) is the absorption at time zero[70–72]. If two exponentials were required to explain the results, the data were fit to a simple sum of two exponentials using the equation described above. The Pfr→Pr photoconversion rate constant for phyB+PIF6β was 8.57 min^{-1}, which is slightly slower than those of phyB alone (9.59 min^{-1}) and phyB+PIF6β$^{3\ mut}$ (9.29 min^{-1}). Moreover, the thermal reversion rate constant for phyB+PIF6β was 0.0048 min^{-1}, which is significantly slower than those of phyB (0.175 min^{-1}) and phyB+PIF6β$^{3\ mut}$ (0.114 min^{-1}). All data were processed and fitted using GraphPad Prism 9.5 and Origin 2024. The rate constants for these samples are presented as the mean±standard deviation.

Supplementary Movie:

Movie S1: Proposed working model for PIF6-mediated red light signal transduction of plant phytochrome B.
